# Broadly Reactive H2 Hemagglutinin Vaccines Elicit Cross-Reactive Antibodies in Ferrets Preimmune to Seasonal Influenza A Viruses

**DOI:** 10.1128/mSphere.00052-21

**Published:** 2021-03-10

**Authors:** Z. Beau Reneer, Amanda L. Skarlupka, Parker J. Jamieson, Ted M. Ross

**Affiliations:** a Center for Vaccines and Immunology, University of Georgia, Athens, Georgia, USA; b Department of Infectious Diseases, University of Georgia, Athens, Georgia, USA; University of Michigan-Ann Arbor

**Keywords:** COBRA, pandemic, ferrets, influenza, H2N2, preimmunity

## Abstract

Influenza vaccines have traditionally been tested in naive mice and ferrets. However, humans are first exposed to influenza viruses within the first few years of their lives. Therefore, there is a pressing need to test influenza virus vaccines in animal models that have been previously exposed to influenza viruses before being vaccinated. In this study, previously described H2 computationally optimized broadly reactive antigen (COBRA) hemagglutinin (HA) vaccines (Z1 and Z5) were tested in influenza virus “preimmune” ferret models. Ferrets were infected with historical, seasonal influenza viruses to establish preimmunity. These preimmune ferrets were then vaccinated with either COBRA H2 HA recombinant proteins or wild-type H2 HA recombinant proteins in a prime-boost regimen. A set of naive preimmune or nonpreimmune ferrets were also vaccinated to control for the effects of the multiple different preimmunities. All of the ferrets were then challenged with a swine H2N3 influenza virus. Ferrets with preexisting immune responses influenced recombinant H2 HA-elicited antibodies following vaccination, as measured by hemagglutination inhibition (HAI) and classical neutralization assays. Having both H3N2 and H1N1 immunological memory regardless of the order of exposure significantly decreased viral nasal wash titers and completely protected all ferrets from both morbidity and mortality, including the mock-vaccinated ferrets in the group. While the vast majority of the preimmune ferrets were protected from both morbidity and mortality across all of the different preimmunities, the Z1 COBRA HA-vaccinated ferrets had significantly higher antibody titers and recognized the highest number of H2 influenza viruses in a classical neutralization assay compared to the other H2 HA vaccines.

**IMPORTANCE** H1N1 and H3N2 influenza viruses have cocirculated in the human population since 1977. Nearly every human alive today has antibodies and memory B and T cells against these two subtypes of influenza viruses. H2N2 influenza viruses caused the 1957 global pandemic and people born after 1968 have never been exposed to H2 influenza viruses. It is quite likely that a future H2 influenza virus could transmit within the human population and start a new global pandemic, since the majority of people alive today are immunologically naive to viruses of this subtype. Therefore, an effective vaccine for H2 influenza viruses should be tested in an animal model with previous exposure to influenza viruses that have circulated in humans. Ferrets were infected with historical influenza A viruses to more accurately mimic the immune responses in people who have preexisting immune responses to seasonal influenza viruses. In this study, preimmune ferrets were vaccinated with wild-type (WT) and COBRA H2 recombinant HA proteins in order to examine the effects that preexisting immunity to seasonal human influenza viruses have on the elicitation of broadly cross-reactive antibodies from heterologous vaccination.

## INTRODUCTION

The 1957 “Asian Influenza” pandemic was caused by an H2N2 influenza virus resulting in an estimated one to two million deaths worldwide (1). This novel H2N2 influenza virus was the result of a reassortment event between a human H1N1 influenza virus and an avian H2N2 influenza virus (2). This novel H2N2 influenza virus contained the HA, NA, and PB1 genome segments from an avian H2N2 influenza virus and the other five genome segments from a human H1N1 influenza virus (3). The 1889 influenza pandemic may also have been caused by an H2N2 influenza virus (4). Therefore, as at least one of the last five influenza pandemics was caused by an influenza virus from the H2N2 subtype, it is likely that a future pandemic will be caused by an H2N2 influenza virus.

H2 influenza viruses have not been as extensively studied as other influenza A virus subtypes, such as H1, H3, H5, or H7. While H2 influenza viruses have been isolated numerous times from wild avian species and domestic poultry (5–10), there have been no known viral infections of humans since the 1960s. However, in 2006, a novel H2 influenza virus was isolated from two separate swine farms in Missouri (11). This swine-derived H2N3 influenza virus has been shown to cause severe disease in both mice and ferrets (12). The H2 hemagglutinin (HA) is also capable of obtaining a multibasic cleavage site and remaining functional, which could have dire implications for both humans and poultry in the future (13).

The goal of this study was to evaluate how memory immune responses to previous influenza virus infections affect broadly reactive HA-based vaccinations. To develop broadly reactive influenza virus vaccines, our group has used the methodology for enhanced antigen design, termed computationally optimized broadly reactive antigen (COBRA) to design hemagglutinin (HA) immunogens for the H1, H3, and H5 influenza subtypes (14–21). This process utilizes multiple rounds of layered consensus building to generate influenza virus vaccine HA antigens that are capable of eliciting broadly reactive HA antibodies, which can protect against both seasonal and pandemic influenza strains that have undergone genetic drift (17, 18, 21). These vaccine antigens also inhibit viral infection and virus-induced pathogenesis in mice, ferrets, and nonhuman primates (16, 22–24). Using the consensus layering approach of COBRA design, H2 COBRA HA vaccines were previously developed and characterized (25).

Humans have been infected with different types of influenza viruses throughout their lives (3). Additionally, different subtypes have circulated as seasonal influenza viruses over the past 100 years (3). A person’s history of influenza virus infections has an effect on future influenza vaccinations and infections (26–28). The HA subtype from the first influenza virus infection influences the susceptibility of an individual to subsequent influenza virus infections from other subtypes (27). Therefore, a broadly cross-reactive H2 influenza virus vaccine should be evaluated in animals previously infected or “preimmune” to different influenza virus subtypes.

For this study, Fitch ferrets were infected with different combinations of human isolated H1N1 and H3N2 influenza viruses. These two influenza virus subtypes are the only influenza A viruses that have circulated in the human population since 1968 and would therefore be reflective of the majority of individuals alive today. The H1N1 infections included both a seasonal (before 2009) and a pandemic H1N1 virus (2009 to the present), since individuals alive today who are over the age of 11 would have been exposed to both types of H1N1 influenza viruses. The H1N1 viruses used in this study were Singapore/6/1986 (Sing/86) and California/07/2009 (CA/09), respectively. The H3N2 influenza viruses used to establish preimmunity were either Sichuan/2/1987 (Sich/87) or Panama/2007/1999 (Pan/99). Additionally, the influenza virus preimmunity of individuals alive today would include individuals infected with H1N1 influenza viruses followed by H3N2 influenza viruses and vice versa. Finally, a “nonpreimmune” or “naive preimmune” group was included as a control for the vaccines alone. An H2N2 preimmune group was also included as a pseudo “positive control” group since previous studies have shown that imprinting ferrets with a specific subtype of influenza virus followed by vaccination with another antigenically distinct influenza virus of the same subtype induces expansive intrasubtype antibodies (29). Two antigenically distinct H2N2 avian influenza viruses were used to establish the H2N2 preimmunity because of the restraints of housing ferrets infected with biosafety level 3 (BSL3) human H2N2 influenza viruses in high-level containment for several months.

After preimmunity was established, two H2 COBRA HA vaccines (Z1 and Z5) were used to vaccinate the ferrets. Protective immune responses elicited by the Z1 and Z5 COBRA HA vaccines were compared to the elicited response in preimmune ferrets vaccinated with wild-type H2 HA proteins. The Z1 COBRA HA-vaccinated preimmune ferrets showed more broadly cross-reactive antibody responses to a panel of H2 influenza viruses across each of the six preimmune immune groups compared to ferrets vaccinated with either of the two wild-type H2 HA vaccines. Therefore, the Z1 COBRA HA would be an ideal vaccine for use in individuals regardless of their previous exposure to influenza A viruses.

## RESULTS

### Vaccination of ferrets with preexisting influenza virus immunity.

Fitch ferrets (*n* = 20) were made preimmune with one of three influenza virus subtypes. The H2N2 preimmunity virus used for infection was either Chk/PA/04 or Qu/RI/16. The H3N2 virus used for infection was either Sich/87 or Pan/99. The H1N1 viruses used for infection were both Sing/86 and CA/09 to represent both seasonal and pandemic H1N1 influenza viruses. After each influenza virus infection, the ferrets were allowed to recover for at least 60 days. Approximately 60 days after the final infection, ferrets had seroconverted to the infection strains with an average HAI titer greater than 1:40 (Fig. S6 in the supplemental material). The H1N1 alone and H3N2-H1N1 and H1N1-H3N2 preimmune groups were then infected with their second virus and allowed to recover for an additional 60 days. The H1N1-H3N2 and the H3N2-H1N1 preimmune groups were then infected with their third virus and allowed to recover for an additional 60 days.

**FIG 1:**
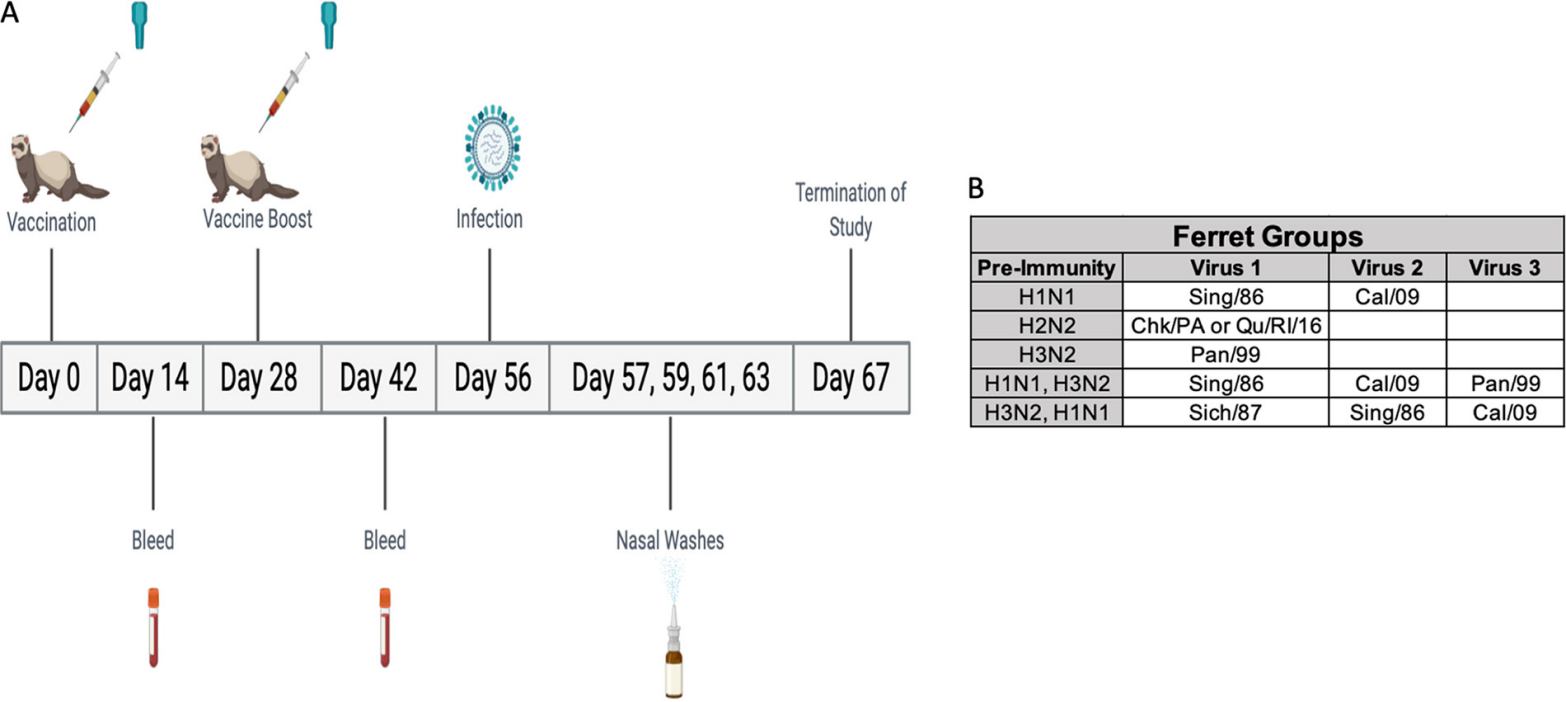
Study outline. A timeline of the procedures following the establishment of preimmunity are listed in panel A. Ferrets were vaccinated at day 0 and day 28. Bleeds were taken at day 14 and day 42. Ferrets were then infected on day 56 and nasal washes were taken on days 57, 59, 61, and 63. The study was terminated on day 67. The viruses used to establish different preimmunities are listed in panel B.

Sixty days after the preimmune groups’ final viral infection, the ferrets were vaccinated with 15 μg of either wild-type (Mal/NL/01 or Mal/WI/08) or COBRA (Z1 or Z5) H2 recombinant HA (rHA) proteins (Fig. 1). A comparison of the amino acids in the antigenic sites of wild-type and COBRA HA sequences is shown in Fig. S1. Four ferrets from each of the preimmune groups received the same vaccine (five vaccines with four ferrets each equates to 20 ferrets per preimmunity group). The mock-vaccinated group was vaccinated with phosphate-buffered saline (PBS) and adjuvant. At 4 weeks after the initial vaccination, the ferrets were vaccinated again with 15 μg of the same antigen as the first vaccination. All of the ferrets were bled 2 weeks after each of the vaccinations.

### Weight loss and survival.

**FIG 2:**
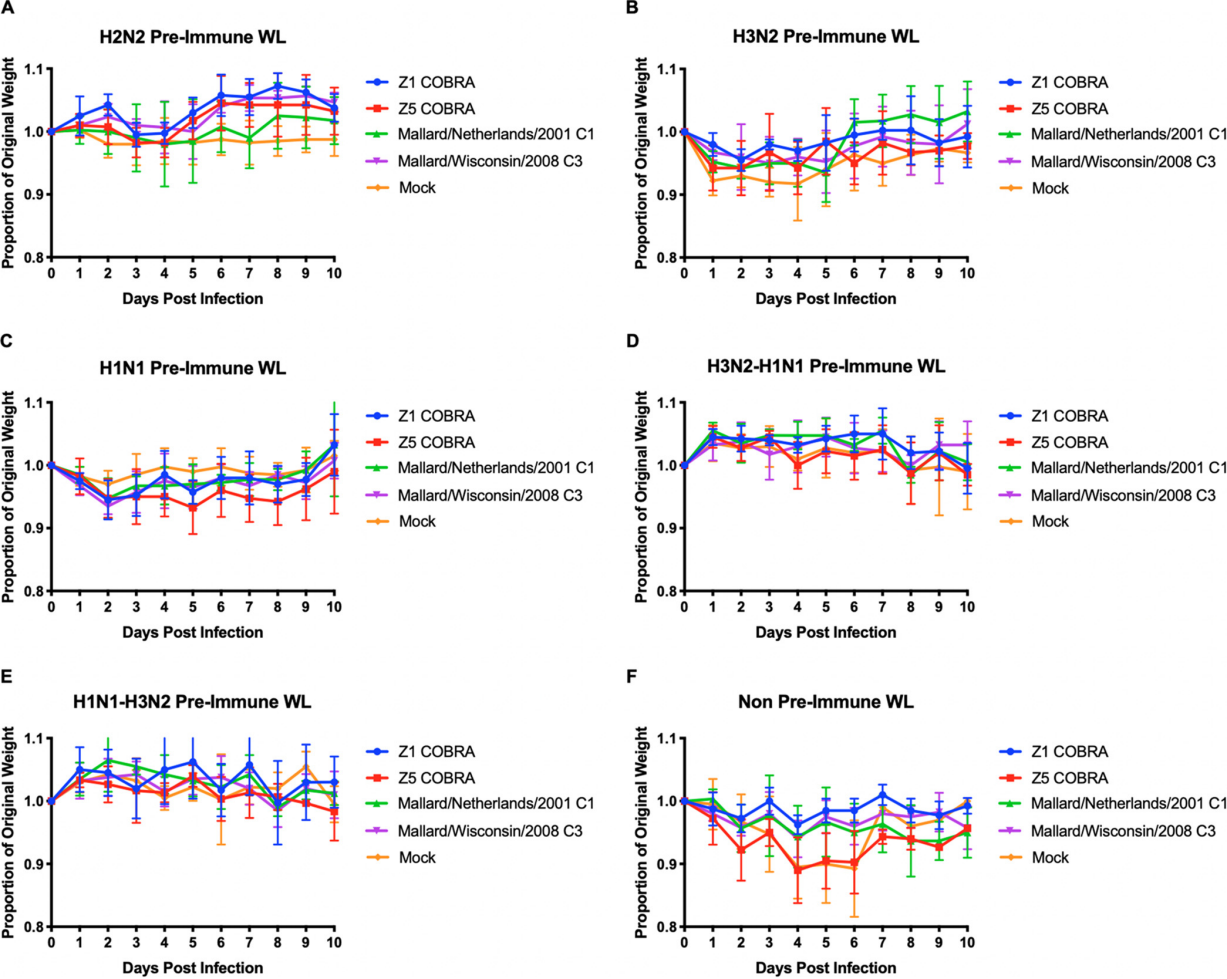
Weight loss of viral-challenged ferrets. Preimmune ferrets vaccinated with wild-type or COBRA rHA vaccines were challenged with Sw/MO/06. Average weight loss was recorded for each vaccine up to 10 days after infection in the H2N2 preimmunity (A), H3N2 preimmunity (B), H1N1 preimmunity (C), H3N2-H1N1 preimmunity (D), H1N1-H3N2 preimmunity (E), and nonpreimmune (F) groups. Error bars represent standard mean error.

Four weeks after the second vaccination, ferrets were infected with the H2N3 clade-3, Sw/MO/06 virus (1e + 6 PFU/ml) (Fig. 1). In the H2N2 preimmune group, there was no significant weight loss after viral challenge and none of the ferrets died (Fig. 2A). In the H3N2 preimmune group, the ferrets in each of the different H2 rHA vaccination groups had an average weight loss of less than 5%, while the mock-vaccinated H3N2 preimmune ferrets reached a peak of 8% average weight loss on day 4 postinfection (Fig. 2B). The Z5-vaccinated ferrets in the H1N1 preimmune group reached a peak average weight loss of 6% on day 5 postinfection. None of the other vaccination groups (Mal/NL/01, Mal/WI/08, or Z1 COBRA) had an average weight loss of greater than 5% in the H1N1 preimmune group (Fig. 2C). The H3N2-H1N1 preimmune ferrets, as well as the H1N1-H3N2 preimmune ferrets, had no detectable average weight loss across any of the four vaccination groups or the mock-vaccinated group after challenge (Fig. 2D and E).

The nonpreimmune ferrets did not have any influenza infection prior to vaccination. Both the Z5- and mock-vaccinated ferrets reached a peak average weight loss of >10% by day 4 postinfection (Fig. 2F). The Mal/NL/01- and Mal/WI/08-vaccinated groups reached a peak average weight loss of ∼5% on day 4 postinfection. The Z1-vaccinated group had a peak average weight loss of ∼3% on day 4 postinfection. The Mal/NL/01-, Mal/WI/08-, and Z1-vaccinated ferrets all survived until the end of the study. The mock- and Z5-vaccinated groups had significantly more weight loss than the Z1-vaccinated ferrets on days 4, 5, and 6 (*P* < 0.01 for mock and *P* < 0.05 for Z5). The mock- and Z5-vaccinated groups also had significantly more weight loss than the Mal/WI/08-vaccinated ferrets on day 5 (*P* < 0.05). These were the only statistically significant differences in weight loss between vaccination groups in any of the preimmune groups.

Only the nonpreimmune and H3N2 preimmune groups experienced mortality. For the nonpreimmune group, one of the Z5-vaccinated ferrets reached humane endpoint by day 6 postinfection (Table 1). Also in the nonpreimmune group, three of the four ferrets in the mock-vaccinated group reached humane endpoint by day 6 postinfection (Table 1). In the H3N2 preimmune group, one ferret in the mock-vaccinated group had to be euthanized on day 5 postinfection (Table 1).

**TABLE 1:** Ferret survival percentage^*a*^

Preimmunity	Vaccine	% Survival after Sw/MO/06 challenge
H2N2	Mal/NL/01	100
H2N2	Mal/WI/08	100
H2N2	Z1	100
H2N2	Z5	100
H2N2	Mock	100
H3N2	Mal/NL/01	100
H3N2	Mal/WI/08	100
H3N2	Z1	100
H3N2	Z5	100
H3N2	Mock	75
H1N1	Mal/NL/01	100
H1N1	Mal/WI/08	100
H1N1	Z1	100
H1N1	Z5	100
H1N1	Mock	100
H3N2, H1N1	Mal/NL/01	100
H3N2, H1N1	Mal/WI/08	100
H3N2, H1N1	Z1	100
H3N2, H1N1	Z5	100
H3N2, H1N1	Mock	100
H1N1, H3N2	Mal/NL/01	100
H1N1, H3N2	Mal/WI/08	100
H1N1, H3N2	Z1	100
H1N1, H3N2	Z5	100
H1N1, H3N2	Mock	100
Mock	Mal/NL/01	100
Mock	Mal/WI/08	100
Mock	Z1	100
Mock	Z5	75
Mock	Mock	25

a The survival rate among ferrets in each of the vaccination groups across all of the preimmunities. The columns correspond to the preimmunity, the vaccine, and the survival percentage after challenge with A/Swine/Missouri/4296424/2006 (Sw/MO/06) (H2N3). Each row is a vaccination group with its preimmunity followed by the survival percentage following viral challenge. Ferret groups contained either *n* = 3 or *n* = 4 ferrets.

### Viral titers.

**FIG 3:**
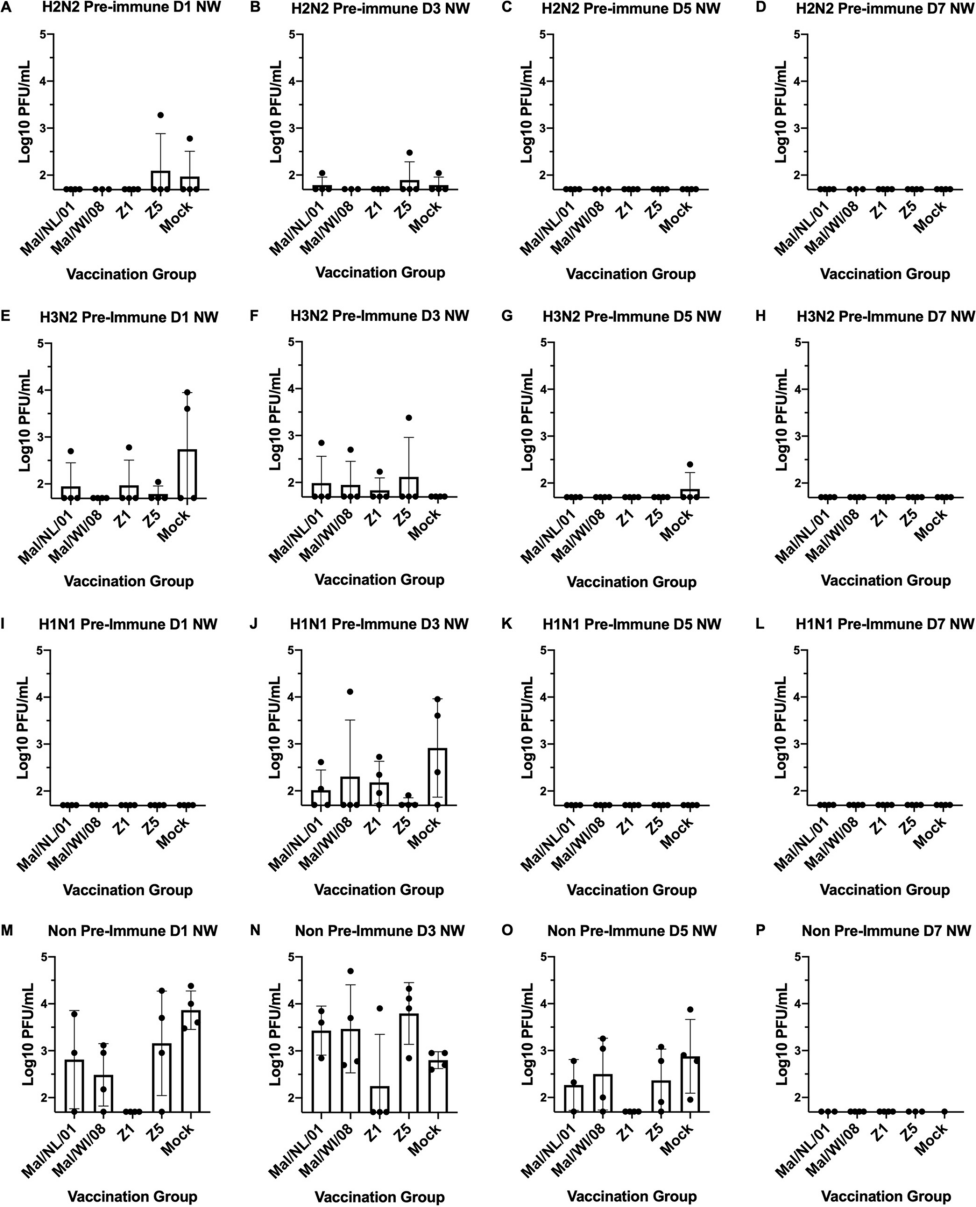
Viral nasal wash titers. Nasal washes were performed on days 1, 3, 5 and 7 postinfection. The titers are recorded as log_10_ PFU/ml. The H2N2 preimmune ferrets are shown in panels A to D. The H3N2 preimmune ferrets are shown in panels E to H. The H1N1 preimmune ferrets are shown in panels I to L. The nonpreimmune ferrets are shown in panels M to P. The height of the bars shows the mean while the error bars represent mean standard error.

In the H2N2 preimmune group, one ferret in both the Z5- and mock-vaccinated groups had viral titers of ∼1.0e + 3 PFU/ml on day 1. One ferret each of the Mal/NL/01-, Z5-, and mock-vaccinated groups had detectable viral titers in their nasal washes at day 3. There were no detectable viral titers in any ferret in the day 5 or day 7 nasal washes (Fig. 3A to D). In the H3N2 preimmune group, one ferret in each of the Mal/NL/01, Z1, and Z5 vaccination groups had viral titers on day 1 (between 1e + 2 and 1e + 3) (Fig. 3E). The mock-vaccinated group had two ferrets with viral titers around 1e + 4, while the Mal/WI/08 ferrets had no detectable titers. On day 3 postinfection, one ferret in each of the vaccination groups besides the mock vaccination group had viral titers between 5e + 2 and 5e + 3 PFU/ml. The mock-vaccinated ferrets had no detectable titers on day 3 (Fig. 3F). On day 5 postinfection, only one ferret in the mock-vaccinated group had detectable viral titers (Fig. 3G). This mock-vaccinated ferret also reached humane endpoint on day5 (Table 1). None of the H3N2 preimmune ferrets had detectable viral titers on day 7 postinfection (Fig. 3H).

None of the ferrets in the H1N1 preimmune group had detectable viral titers in their nasal washes on days 1, 5, or 7 postinfection (Fig. 3I, K to L). All five of the vaccination groups had at least one ferret with detectable viral titers in their nasal washes on day 3. The mock-vaccinated ferrets had the highest average viral titers (∼1e + 3 PFU/ml), while the other four vaccination groups had average viral titers between 5e + 1 and 5e + 2 PFU/ml (Fig. 3J).

In the nonpreimmune group, multiple ferrets in Mal/NL/01, Mal/WI/08, Z5, and mock vaccination groups all had detectable viral titers in their day 1 nasal washes, with multiple ferrets in each of the vaccination groups having ≥3e + 1 viral titers. None of the ferrets in the Z1 vaccination group had detectable viral titers on day 1 postinfection (Fig. 3M). On day 3 postinfection, the average viral titers for the Mal/NL/01-, Mal/WI/08-, Z5-, and mock-vaccinated ferrets all had average viral titers between ∼1e + 3 and 1e + 4. One ferret in the Z1 vaccination group had detectable viral titers on day 3 postinfection (Fig. 3N). On day 5 postinfection, multiple ferrets in the Mal/NL/01-, Mal/WI/08-, Z5-, and mock-vaccinated groups had viral titers >1e + 2, with the mock-vaccinated ferrets having the highest average viral titer of ∼1e + 3. None of the ferrets in the Z1 vaccination group had detectable viral titers on day 5 postinfection (Fig. 3O). None of the surviving ferrets had detectable viral titers in their nasal washes on day 7 postinfection (Fig. 3P). None of the ferrets in the H3N2-H1N1 preimmune or the H1N1-H3N2 preimmune groups had any detectable viral titers in their nasal washes on any day (Fig. S7). Of all the time points and preimmunity backgrounds, there was no significant difference between the vaccinated groups and the mock groups except for nonpreimmune day 1 NW, where Z1 was significantly lower than the mock group with a *P* value of 0.0078 using a one-way ANOVA plus Tukey’s test.

A three-way ANOVA looking at the main effects of vaccine received, the ferret preimmunity, and the day of the nasal wash indicated that overall, when adjusting for preimmunity and day postinfection, that the mean viral nasal wash titers of the Z1 COBRA was significantly lower than that of the mock-vaccinated group by 0.322 log 10 viral titer (*P* adjusted < 0.001). Furthermore, the Z1 COBRA also had a titer of 0.219 log 10 lower after adjustment compared to the Z5 COBRA (*P* adjusted = 0.039) (Fig. S2). Only the nonpreimmune ferrets were significantly different from the other preimmunities after controlling for vaccine received and day postinfection (Fig. S2). All other preimmunities had nonsignificant mean viral titers. When comparing the day postinfection, day 1 and day 3 were not significantly different, but day 5 had lower viral titers compared to either day 3 or day 1.

### Hemagglutination-inhibition (HAI) antibodies.

**FIG 4:**
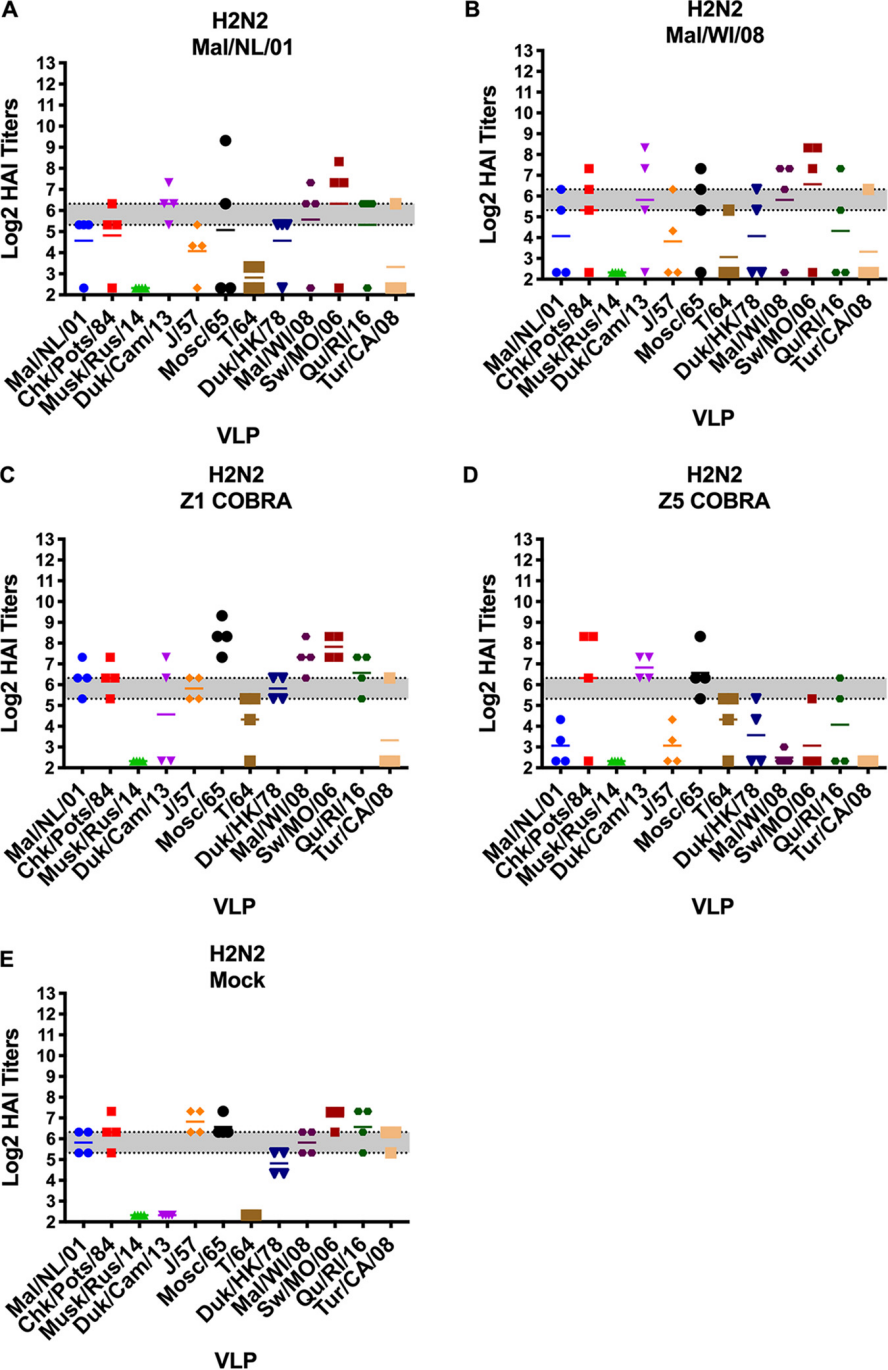
Cross-reactive antibody responses on day 0 (prime vaccination) in H2N2 preimmune ferrets. HAI titers are given for each vaccine group in the H2N2 preimmunity. Serum from each ferret was obtained on the day of prime vaccination (60 days postinfection) and tested against VLPs expressing 12 WT H2 HA sequences. Dotted lines indicate 1:40 and 1:80 HAI titers, respectively. The VLP panel is composed of: clade-1 HAs (Mallard/Netherlands/2001, Chicken/Potsdam/1984, Muskrat/Russia/2014, and Duck/Cambodia/2013), clade-2 HAs (Duck/Hong Kong/1978, Taiwan/1/1964, Moscow/1019/1965, and Japan/305/1957), and clade-3 HAs (Mallard/Wisconsin/2008, Swine/Missouri/2006, Quail/Rhode Island/2016, and Turkey/California/2008). Error bars represent standard mean errors.

The HAI titers varied greatly between the preimmune groups. The H2N2 preimmune ferrets were the only preimmune group to have HAI titers to virus-like particles (VLPs) in the H2 panel on the day of prime vaccination (Fig. 4). Ferrets in all five of the vaccination groups had average HAI titers above 1:40 to multiple VLPs in the H2 panel, but none of the vaccination groups had detectable titers to all 12 of the H2 HA VLPs (Fig. 4).

**FIG 5:**
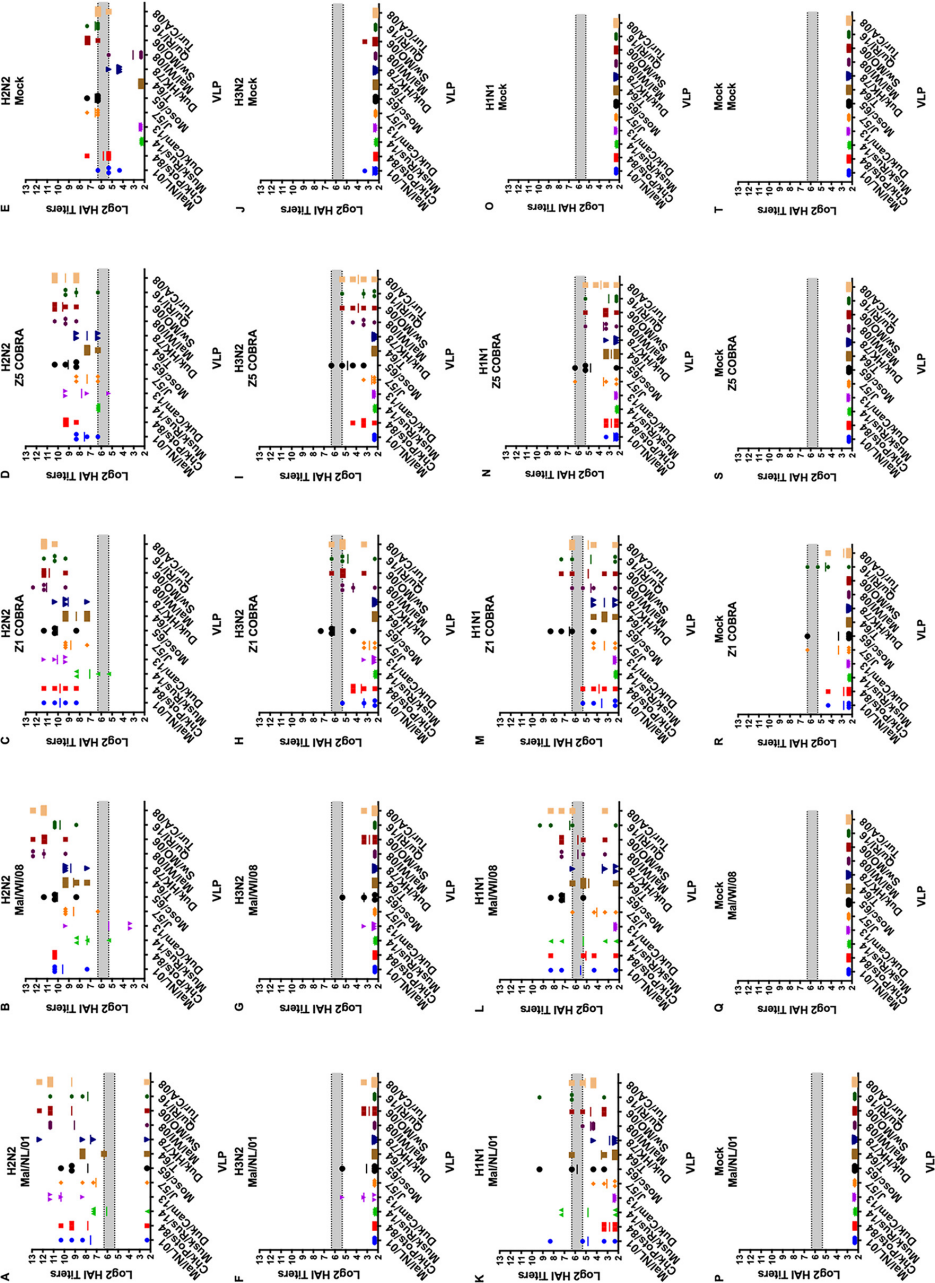
Antibody cross-reactivity of H2N2, H1N1, H3N2, and nonpreimmune groups on day 14 post-prime vaccination. HAI titers are given for each vaccine group 14 days after the first vaccine in the H2N2 preimmunity (A to E), H3N2 preimmunity (F to J), H1N1 preimmunity (K to O), and nonpreimmune (P to T) groups. Serum from each ferret was obtained on day 14 after the first vaccination and tested against VLPs expressing 12 WT H2 HA sequences. Dotted lines indicate 1:40 and 1:80 HAI titers, respectively. The VLP panel is composed of: clade-1 HAs (Mallard/Netherlands/2001, Chicken/Potsdam/1984, Muskrat/Russia/2014, and Duck/Cambodia/2013), clade-2 HAs (Duck/Hong Kong/1978, Taiwan/1/1964, Moscow/1019/1965, and Japan/305/1957), and clade-3 HAs (Mallard/Wisconsin/2008, Swine/Missouri/2006, Quail/Rhode Island/2016, and Turkey/California/2008). Error bars represent standard mean errors.

After the first vaccination, the H2N2 preimmune ferrets had a geometric mean HAI titer of ≥1:40 to all 12 of the VLPs in the panel excluding the mock vaccination group (Fig. 5A to E). The H3N2 preimmune ferrets had low HAI titers to the H2 VLPs after the first vaccination (Fig. 5F to J). Only the Z1-vaccinated ferrets had a geometric mean HAI titer of ≥1:40 to more than one H2 VLP (Fig. 5H). The mock-vaccinated ferrets in H1N1 preimmune group did not have detectable HAI titers to any of the H2 VLPs after the first vaccination. The Mal/NL/01-, Mal/WI/08-, and Z1-vaccinated ferrets had geometric mean HAI titers of ≥1:40 against multiple H2 VLPs after the first vaccination (Fig. 5K, L, N, and O). The Z5-vaccinated H1N1 preimmune ferrets only had geometric mean HAI titers of ≥1:40 against one H2 VLP (Fig. 5M). The nonpreimmune ferrets in the Mal/NL/01, Mal/WI/08, Z5, and mock vaccination groups had no detectable HAI titers after the first vaccination (Fig. 5P, Q, S, and T). The Z1-vaccinated ferrets had some HAI titers after the first vaccination, but none of them reached a geometric mean titer of 1:40 (Fig. 5R).

**FIG 6:**
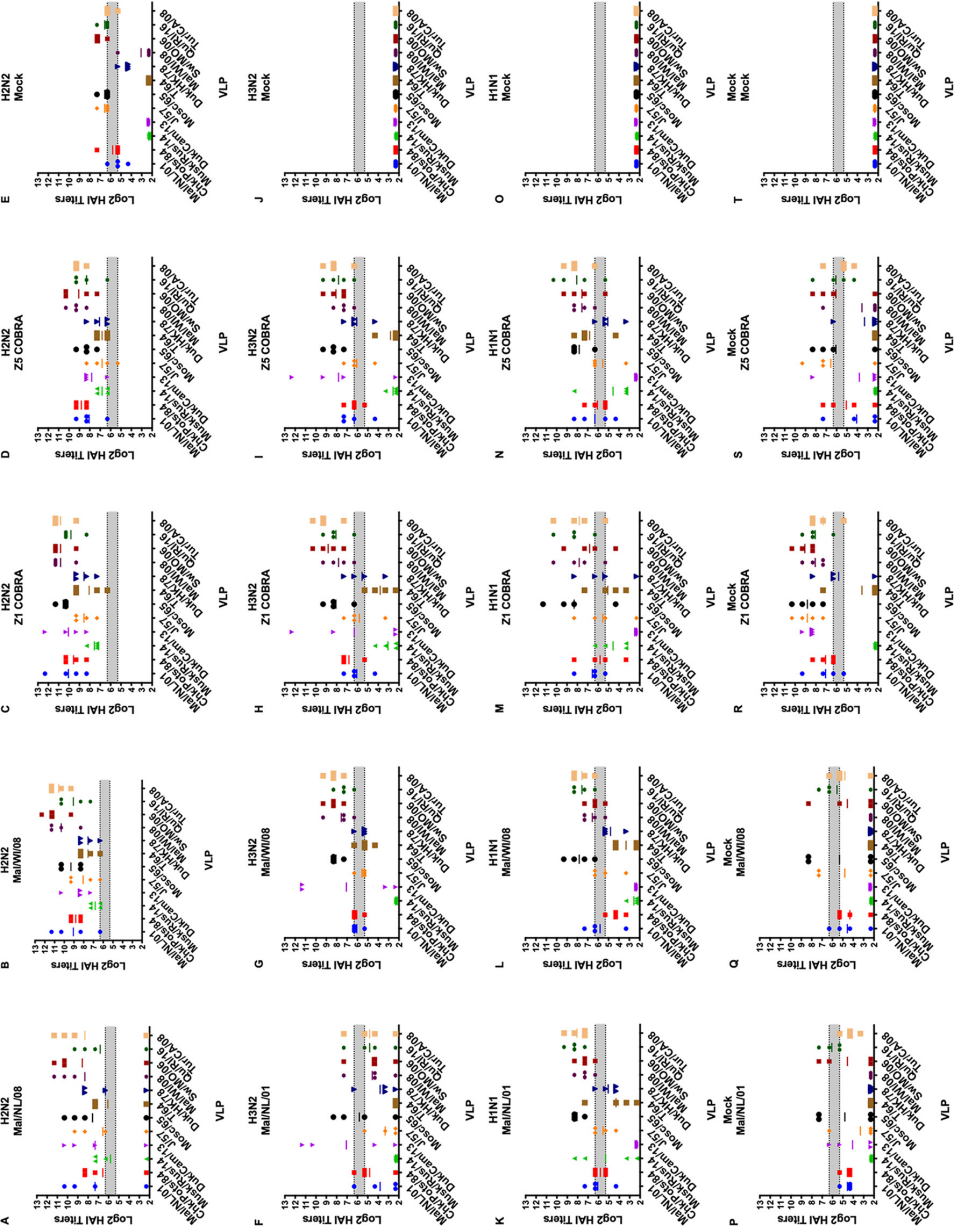
Antibody cross-reactivity of H2N2, H1N1, H3N2, and nonpreimmune groups on day 42 post-prime (day 14 post-boost) vaccination. HAI titers are given for each vaccine group 14 days after the second vaccination in the H2N2 preimmunity (A to E), H3N2 preimmunity (F to J), H1N1 preimmunity (K to O), and nonpreimmune (P to T) groups. Serum from each ferret was obtained on day 42 postvaccination and tested against VLPs expressing 12 WT H2 HA sequences. Dotted lines indicate 1:40 and 1:80 HAI titers, respectively. The VLP panel is composed of: clade-1 HAs (Mallard/Netherlands/2001, Chicken/Potsdam/1984, Muskrat/Russia/2014, and Duck/Cambodia/2013), clade-2 HAs (Duck/Hong Kong/1978, Taiwan/1/1964, Moscow/1019/1965, and Japan/305/1957), and clade-3 HAs (Mallard/Wisconsin/2008, Swine/Missouri/2006, Quail/Rhode Island/2016, and Turkey/California/2008). Error bars represent standard mean errors.

After the second vaccination, the HAI titers for the ferrets in the H2N2 preimmune group did not drastically change (Fig. 6A to E). In the H3N2 preimmune group, HAI titers in the Mal/NL/01-, Mal/WI/08-, Z1-, and Z5-vaccinated ferrets all increased significantly after the second vaccination. This was the only statistically significant increase in average titer using multiple *t* test analyses comparing for the change in each vaccine titer between days 14 and 42 post-prime vaccination. Each of these four vaccination groups had HAI titers of ≥1:40 to seven or more VLPs in the panel. The Z1- and Z5-vaccinated ferrets had geometric mean HAI titers of ≥1:80 to 9 and 10 of the 12 VLPs in the panel, respectively (Fig. 6F to J).

In the H1N1 preimmune ferrets, the HAI titers of all of the vaccination groups besides the mock vaccination group increased after the second vaccination (Fig. 6K to O). After the second vaccination, the Mal/WI/08 vaccination group had geometric mean HAI titers of ≥1:40 to seven of the 12 VLPs in the panel. The Mal/NL/01-, Z1-, and Z5-vaccinated ferrets all had geometric mean HAI titers of >1:40 to 10 or more of the VLPs in the H2 panel. The Mal/NL/01- and the Mal/WI/08-vaccinated ferrets had a geometric mean HAI titer of ≥1:80 to seven and six of the 12 VLPs in the H2 panel, respectively. The Z1- and Z5-vaccinated ferrets had geometric mean HAI titers of ≥1:80 to nine of the 12 VLPs in the H2 panel.

In the nonpreimmune group, the HAI titers in the Mal/NL/01, Mal/WI/08, Z1, and Z5 vaccination groups all increased after the second vaccination (Fig. 6P to T). The Mal/NL/01- and Mal/WI/08-vaccinated ferrets had geometric mean HAI titers of ≥1:40 to 3 and 5 of the VLPs in the H2 panel. The Z1- and Z5-vaccinated ferrets had geometric mean HAI titers of ≥1:40 to 11 and 9 of the VLPs, respectively. The Mal/NL/01-, Mal/WI/08-, and Z5-vaccinated ferrets had geometric mean HAI titers of ≥1:80 to 2, 4, and 5 of the H2 VLPs in the panel, respectively. The Z1-vaccinated ferrets had geometric mean HAI titers of ≥1:80 to 10 of the 12 VLPs in the H2 panel.

**FIG 7:**
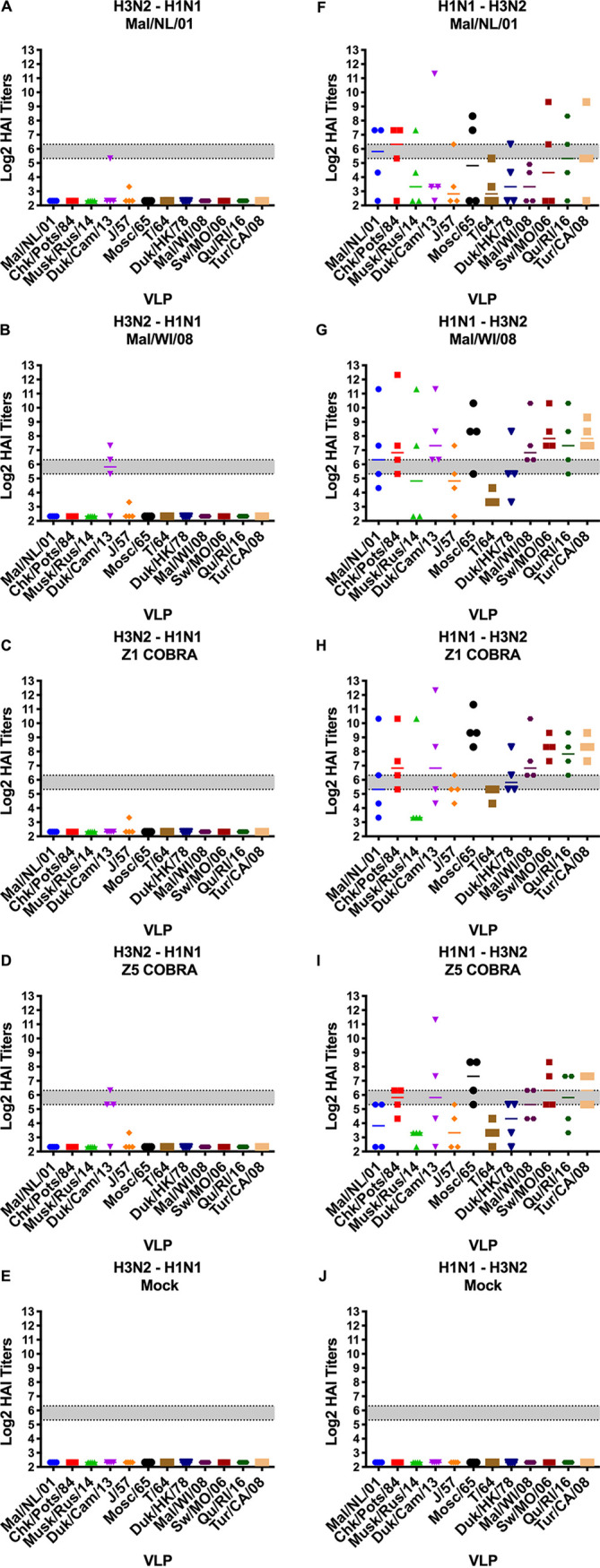
Antibody cross-reactivity of H1N1-H3N2 and H3N2-H1N1 preimmune groups on day 14 post-prime vaccination. HAI titers are given for each vaccine group 14 days after the first vaccination (day 14) in the H1N1-H3N2 preimmunity (A to E) and H3N2-H1N1 preimmunity (F to J) groups. Dotted lines indicate 1:40 and 1:80 HAI titers, respectively. The VLP panel is composed of: clade-1 HAs (Mallard/Netherlands/2001, Chicken/Potsdam/1984, Muskrat/Russia/2014, and Duck/Cambodia/2013), clade-2 HAs (Duck/Hong Kong/1978, Taiwan/1/1964, Moscow/1019/1965, and Japan/305/1957), and clade-3 HAs (Mallard/Wisconsin/2008, Swine/Missouri/2006, Quail/Rhode Island/2016, and Turkey/California/2008). Error bars represent standard mean errors.

The H3N2-H1N1 and the H1N1-H3N2 preimmune groups varied greatly in their HAI responses. The H3N2-H1N1 preimmune group had little to no HAI titers after the first vaccination to any of the VLPs in the panel (Fig. 7A to E). The H1N1-H3N2 preimmune group had multiple ferrets in each vaccination group with detectable HAI titers to each of the 12 VLPs in the panel (Fig. 7F to J). The Z5 and Mal/NL/01 vaccination groups had a geometric mean HAI titer of >1:40 to 7 and 8 of the VLPs in the panel, respectively. The Mal/WI/08 and Z1 vaccination groups had a geometric mean HAI titer of ≥1:40 to 11 and 12 out of the 12 VLPs in the panel, respectively.

**FIG 8:**
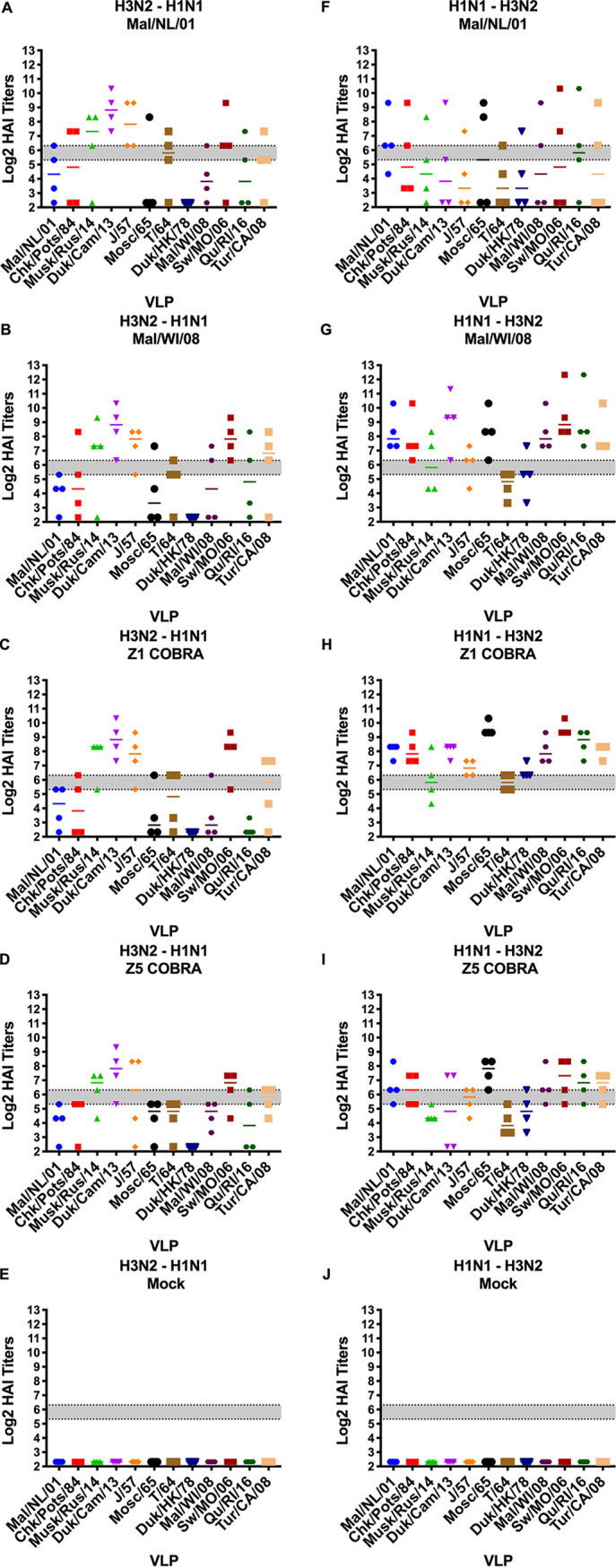
Antibody cross-reactivity of H1N1-H3N2 and H3N2-H1N1 preimmune groups on day 42 post-prime (day 14 post-boost) vaccination. HAI titers are given for each vaccine group 14 days after the second vaccination (day 42) in the H1N1-H3N2 preimmunity (A to E) H3N2-H1N1 preimmunity (F to J) groups. Dotted lines indicate 1:40 and 1:80 HAI titers, respectively. The VLP panel is composed of: clade-1 HAs (Mallard/Netherlands/2001, Chicken/Potsdam/1984, Muskrat/Russia/2014, and Duck/Cambodia/2013), clade-2 HAs (Duck/Hong Kong/1978, Taiwan/1/1964, Moscow/1019/1965, and Japan/305/1957), and clade-3 HAs (Mallard/Wisconsin/2008, Swine/Missouri/2006, Quail/Rhode Island/2016, and Turkey/California/2008). Error bars represent standard mean errors.

After the second vaccination, the Z5- and Z1-vaccinated ferrets in the H3N2-H1N1 preimmune group had HAI titers of ≥1:40 to 5 and 6 of the VLPs in panel, respectively. The Mal/NL/01- and the Mal/WI/08-vaccinated ferrets had HAI titers of ≥1:40 to 9 and 10 of the 12 VLPs in the panel, respectively (Fig. 8A to E). After the second vaccination for the H1N1-H3N2 preimmune group, the Z5-vaccinated ferrets had a geometric mean HAI titer of ≥1:40 to 10 of the VLPs, while the Z1-vaccinated ferrets had a geometric mean HAI titer of ≥1:40 to all 12 of the VLPs in the panel. The Mal/NL/01 and Mal/WI/08 vaccination groups had a geometric mean HAI titer of ≥1:40 to 11 of the VLPs in the panel (Fig. 8F to J).

After controlling for the main effects of the vaccine received, preexisting immunity, and HAI VLP, the Z1 COBRA vaccine had a significantly higher overall log_2_ mean HAI titer compared to the other vaccine groups using an ANOVA with Tukey adjustment method. The Z5 COBRA and Mal/WI/08 vaccines were not significantly different from each other, and the Mal/NL/01 vaccine titers were significantly lower than the other vaccine groups. For preimmunity, the ferrets with an H2N2 background had a significantly higher mean titer than compared to the H1N1, H3N2, and H1N1-H3N2 group, which were not significantly different from each other. The H3N2-H1N1 preimmunity overall mean was not significantly different from the mock preimmunity mean titer.

### Neutralization assays.

Serum was collected from the ferrets after the second vaccination and pooled in equal amounts for the neutralization assay. The Mal/NL/01-, Mal/WI/08-, and Z1-vaccinated ferrets with the H2N2 preimmunity all had neutralizing antibodies titers of ≥1:350 to 5 of the 7 influenza viruses in the neutralization panel (Table 2). The Z5-vaccinated ferrets had neutralizing antibodies titers of ≥1:350 to 6 of the 7 influenza viruses. The H3N2 preimmune ferret groups all had neutralization titers lower than 1:200 to all of the influenza viruses in panel except for Sw/MO/06, where the Mal/NL/01-, Mal/WI/08-, Z1-, and Z5-vaccinated ferrets all had titers of >1:450. In the H1N1 preimmune group, the Mal/NL/01 and Z5 vaccine groups had neutralization titers of >1:100 to 2 of the 7 influenza viruses in the panel, while the Mal/WI/08 and Z1 vaccine groups had neutralization titers of >1:100 for 4 and 5 of the influenza viruses, respectively. The Mal/NL/01- and Z5-vaccinated ferrets in the H3N2-H1N1 preimmune group had neutralization titers of >1:200 to 2 of the 7 influenza viruses in the H2Nx virus panel. The Mal/WI/08-vaccinated ferrets had neutralization titers of >1:200 only to the Sw/MO/06 H2N3 influenza virus. The Z1-vaccinated ferrets had titers of >200 to 5 of the 7 influenza viruses. For the H1N1-H3N2 preimmune group, the Mal/NL/01-, Mal/WI/08-, Z1-, and Z5-vaccinated ferrets all had neutralization titers of >1:150 to 4 of the 7 influenza viruses. For the nonpreimmune group, the Mal/NL/01-, Mal/WI/08-, Z1-, and Z5-vaccinated ferrets all had neutralization titers of >1:350 to the Sw/MO/06 influenza virus. The Z1-vaccinated ferrets had neutralization titers of >1:150 to 3 of the 6 other influenza viruses in the panel, while the Z5-vaccinated ferrets had neutralization titers of >1:150 to one other influenza virus in the panel.

**TABLE 2:** Ferret neutralization titers on day 42 post-prime sera (day 14 post-boost)^*a*^

Preimmunity	Vaccine	Significance group	Chk/pots/84	Chk/PA/04	For/57	T/64	Duk/HK/78	Sw/MO/06	Mall/MN/08
H2N2	Mal/NL/01	a	640	380	48	34	640	640	452
H2N2	Mal/WI/08	a	640	537	48	56	640	640	640
H2N2	Z1	a	640	640	134	67	640	640	640
H2N2	Z5	a	452	640	40	380	537	640	320
H2N2	Mock	b	95	156	8	24	48	537	95
H3N2	Mal/NL/01	b	17	7	5	20	12	452	17
H3N2	Mal/WI/08	ab	48	67	5	28	24	640	67
H3N2	Z1	a	190	56	7	40	67	640	95
H3N2	Z5	ab	113	113	5	20	34	640	48
H3N2	Mock	c	5	7	5	5	5	6	6
H1N1	Mal/NL/01	b	113	20	5	95	28	537	24
H1N1	Mal/WI/08	ab	113	40	6	537	34	640	226
H1N1	Z1	a	537	269	20	80	269	640	269
H1N1	Z5	ab	134	48	10	20	80	640	12
H1N1	Mock	c	8	6	5	5	5	5	5
H3N2, H1N1	Mal/NL/01	ac	269	160	28	34	95	640	134
H3N2, H1N1	Mal/WI/08	a	113	113	10	34	67	640	134
H3N2, H1N1	Z1	c	640	380	28	160	537	640	226
H3N2, H1N1	Z5	ac	226	160	17	48	160	640	160
H3N2, H1N1	Mock	b	5	5	7	5	5	6	6
H1N1, H3N2	Mal/NL/01	a	640	640	34	5	80	640	640
H1N1, H3N2	Mal/WI/08	a	537	269	40	40	80	640	380
H1N1, H3N2	Z1	a	226	640	67	113	95	640	537
H1N1, H3N2	Z5	a	269	269	20	40	113	640	190
H1N1, H3N2	Mock	b	5	6	5	5	5	5	5
Mock	Mal/NL/01	b	34	14	5	10	10	640	20
Mock	Mal/WI/08	ab	12	17	5	5	7	640	20
Mock	Z1	c	537	190	40	80	226	640	80
Mock	Z5	b	160	28	6	10	56	380	14
Mock	Mock	a	5	5	5	5	5	5	5

a Neutralization titers were obtained from pooled sera. Titers were obtained by taking the geometric mean titer of the replicates for each of the vaccine groups. Each column is a virus that was used in the neutralization assay. Each row is the sera from a vaccination group. The lower limit of detection is 5, while the upper limit of detection is 640. Significance group is determined for each individual preimmunity group. Within one preimmune group, the vaccines’ ability to elicit neutralization titers were compared while controlling for the main effects of virus used in the assay. Vaccination groups within a preimmunity that share a lowercase letter were not significantly different from one another.

## DISCUSSION

Currently, the United States stockpiles prepandemic influenza virus vaccines for both H5 and H7 influenza subtypes (30). While both H5 and H7 influenza viruses have infected humans, there has been little human-to-human transmission of these influenza virus subtypes. Conversely, H2N2 influenza viruses caused the 1957 influenza pandemic (3). It is likely that H2 influenza viruses may cause future pandemics and, therefore, next-generation, broadly reactive universal influenza vaccines should elicit protective immune responses against current and future H2 influenza viruses. Currently, humans develop long-lasting memory cells against seasonal influenza A and B viruses following infection and/or vaccination. However, few studies have investigated the effects of vaccinating individuals for a novel subtype of influenza virus in the presence of preexisting immunity to historical influenza strains. This study addressed the effectiveness of vaccinating ferrets that have preexisting immunity induced by historical influenza A virus (IAV) strains with both WT and COBRA H2 rHA vaccines to stimulate protective immune responses.

IAV naive ferrets were infected individually with H1N1, H2N2, or H3N2 influenza viruses prior to H2 rHA vaccination. Some ferrets were also infected sequentially with H1N1 and H3N2 influenza viruses. Following a single vaccination of the COBRA HA vaccines, ferrets preimmune to group 1 influenza A viruses all had broadly cross-reactive antibodies. This included the ferrets that were administered the H1N1 virus prior to the H3N2 virus. Ferrets infected with H3N2 influenza viruses initially had no cross-reactive HAI titers to H2 influenza viruses after the first vaccination, which was similar to naive ferrets. This result shows that the first subtype of influenza virus that a person is infected with affects their future immune responses to subsequent heterotypic influenza virus vaccinations. This phenomenon, termed “immune imprinting,” has been well-documented for IAV (26–28). These previous studies showed how the IAV that an individual was first infected with seemed to confer protection to other influenza virus subtypes within the same HA evolutionary group. Group 1 influenza virus HA subtypes include H1, H2, H5, and H9, while group 2 influenza virus HA subtypes include H3 and H7 (27, 28). In this study, the broader HAI activity after the prime vaccination in the H1N1 preimmune ferrets compared to the H3N2 preimmune ferrets further support the IAV group-specific immune imprinting theory.

*De novo* immune responses appear more important and are required for the H3N2 (group 2) imprinted ferrets to generate cross-reactive antibodies to H2 influenza viruses than is the case for the H1N1 or H2N2 (group 1) preimmune ferrets. Once the animals were administered a second vaccination, the H3N2 and H3N2-H1N1 preimmune groups had detectable HAI titers, but the titers were 2- to 4-fold lower on average than group 1-imprinted ferrets. The group 2-imprinted ferrets likely have B cells that are highly specific to epitopes on the H3 HA (31). These epitopes are far less similar to the epitopes on H2 HA than the epitopes on H1 HA. Without any similar immune memory, the H3N2 influenza virus-imprinted ferrets would likely need to generate *de novo* B cells to the COBRA H2 HA vaccine (27). Meanwhile, the H1N1 influenza virus-imprinted ferrets have memory B cells that may recognize similar epitopes on the H2 HA proteins. These memory B cells would undergo somatic hypermutation and adapt to the H2 HA epitopes (32). Therefore, sequential infections of influenza viruses may affect the immune responses to future influenza virus infections or vaccinations (27, 28, 31, 32) Since most people under 55 years old will have preexisting memory cells to both H1N1 and H3N2 influenza viruses, a stockpiled H2 influenza virus vaccine will likely need a prime and boost vaccination in order to generate *de novo* immune responses to adequately protect the majority of individuals from a future H2 influenza virus infections.

Mortality in the mock-vaccinated ferrets was absent in all of the preimmune groups with the exception of the naive preimmune ferrets following the H2N3 virus challenge. This result was surprising, since none of the mock-vaccinated ferrets in any of the preimmune groups (with the exception of the H2N2 preimmune ferrets) had substantial HAI or neutralization titers to any of the H2 VLPs or influenza viruses tested in these assays. Given these results, it is likely that other immune mechanisms may be playing a role in protecting ferrets from mortality during the viral challenge. Without H2-specific neutralizing antibodies, it is possible that either nonneutralizing antibodies or T cells are contributing to protection against the Sw/MO/06 H2N3 virus infection (33–36). Nonneutralizing antibodies could be contributing to protection through antibody-dependent cellular cytotoxicity (ADCC) or complement-dependent cytotoxicity (37–39). Both CD4^+^ and CD8^+^ T cells are elicited following infection and they are effective against secondary influenza virus infections (40, 41). There may be epitopes on the HA that could elicit broadly cross-reacting T cells that help clear virally infected cells (42, 43). In addition, influenza virus-elicited T cells responsive to internal gene products, such as nucleoprotein (NP) of both the H1N1 and H3N2 viruses, could recognize epitopes on the internal genes of the Sw/MO/06 challenge virus. Further studies are needed to identify the exact mechanism of protection in these preimmune animal models.

Across all of the preimmune groups, the Z1-vaccinated ferrets had significantly higher cross-reactive H2 antibody titers compared to the other vaccination groups. The Z1-vaccinated ferrets had the highest average HAI titers and recognized more H2 strains than the other vaccines across all of the different preimmunities. The Z1-vaccinated ferrets also had the highest average neutralization titers to more H2 influenza viruses regardless of the preimmune background. The COBRA H2 HA vaccine is likely outperforming the wild-type H2 HA vaccines because they have more diverse epitopes. Higher diversity of epitopes in the COBRA HA would more likely elicit B cells that cross-react across different antigenic sites on the H2 HA. Higher diversity of epitopes is beneficial for vaccinating people who are all preimmune to either H1N1 and/or H3N2 influenza viruses. Vaccinating with a COBRA H2 HA antigen with highly diverse cross-reactive epitopes on a single antigen would increase the likelihood that multiple cross-reactive B cells will be retained in long-term immunological memory.

The Z1 COBRA HA also outperformed the Z5 COBRA and the two wild-type vaccines. Z1 outperforming Z5 was somewhat surprising, since there are only four amino acids that differ between the two HA sequences. However, these four amino acids are spread across three of the seven antigenic sites on the H2 HA molecule (44, 45). It is likely that these mutations are altering the structure of multiple epitopes that are recognized by B cells and resulting in decreased antibody cross-reactive binding to other H2 HA proteins. Therefore, the Z1 COBRA HA would be an ideal vaccine candidate for a future stockpiled H2 influenza virus vaccine to be administered to protect people from a future H2 influenza virus pandemic.

## MATERIALS AND METHODS

### Viruses, recombinant HA proteins, and virus-like particles.

A/Chicken/Potsdam/4705/1984 (Chk/Pots/84) (H2N2) (clade-1), A/Chicken/PA/298101-4/2004 (Chk/PA/04) (H2N2) (clade-1), A/Duck/Hong Kong/273/1978 (Duk/HK/78) (H2N2) (clade-2), A/Mallard/Minnesota/AI08-3437/2008 (Mal/MN/08) (H2N3) (clade-3), A/Swine/Missouri/4296424/2006 (Sw/MO/06) (H2N3) (clade-3), A/Formosa/313/1957 (For/57) (H2N2) (clade-2), and A/Taiwan/1/1964 (T/64) (H2N2) (clade-2) were obtained from either the United States Department of Agriculture (USDA) Diagnostic Virology Laboratory (DVL) in Ames, Iowa, from BEI resources (Manassas, VA, USA), or provided by the laboratory of S. Mark Tompkins (Athens, GA, USA). Each influenza virus was passaged using embryonated chicken eggs except for the Sw/MO/06 virus, which was passaged in MDCK cells. Each influenza virus was harvested from either the eggs or cells and aliquoted into tubes which were stored at −80°C. Each influenza virus was titered using a standard influenza plaque assay as described below.

Recombinant HA (rHA) proteins were expressed using the pcDNA 3.1+ plasmid (Addgene, Watertown, MA). Each HA gene was truncated by removing the transmembrane (TM) domain and the cytoplasmic tail at the 3′ end of the gene (amino acids 527 to 562). The TM domain was determined using the TMHMM Server v. 2.0 website: http://www.cbs.dtu.dk/services/TMHMM/. The HA gene was truncated at the first amino acid prior to the TM domain. A fold-on domain from T4 bacteriophage (46), an Avitag (47), and a 6× histidine tag totaling 477 nucleotides were added to the 3′ end of the HA gene. The pcDNA 3.1+ vectors were then transfected individually into human embryonic kidney (HEK293T) suspension cells using ExpiFectamine 293 transfection reagent (Thermo Fisher Scientific, Waltham, MA, USA) following the manufacturer’s specifications. The supernatants (∼500 ml) were then harvested from the transfected HEK293T cells. Each rHA was then purified from the supernatant using a nickel-agarose column (Thermo Fisher Scientific, Waltham, MA, USA). The rHA proteins were then eluted from the column using 100 mM imidazole (Thermo Fisher Scientific, Waltham, MA, USA). After elution, the proteins were quantified using bicinchoninic assay (BCA) (Thermo Fisher Scientific, Waltham, MA, USA) and stored at −80°C. Recombinant HA proteins produced for this study were A/Mallard/Netherlands/13/2001 (Mal/NL/01), Mallard/Wisconsin/08OS2844/2008 (Mal/WI/08), Z1 COBRA (Z1), and Z5 COBRA (Z5).

For the virus-like particle (VLP) production, adherent human endothelial kidney 293T (HEK-293T) cells were grown in complete Dulbecco’s modified eagles’ medium (DMEM). Once confluent (1 × 10^6^), these cells were transiently transfected for the creation of mammalian virus-like particles (VLPs). Viral proteins were expressed from the pTR600 mammalian expression vectors (48). Influenza virus neuraminidase (A/South Carolina/1/1918; H1N1), the HIV p55 Gag, and HA expression plasmids expressing one of the H2 wild-type or H2 COBRA HA proteins were added to serum-free medium following the Lipofectamine 3000 protocol in a 1:2:1 ratio with a final DNA concentration of 1 μg. Following 72h of incubation at 37°C, supernatants from transiently transfected cells were collected, centrifuged to remove cellular debris, and filtered through a 0.22-μm pore membrane. VLPs were purified and sedimented by ultracentrifugation on a 20% glycerol cushion at 23,500 × *g* for 4 h at 4°C. VLPs were resuspended in phosphate-buffered saline (PBS) and the total protein concentration was determined with the Micro BCA protein assay reagent kit (Pierce Biotechnology, Rockford, IL, USA). Hemagglutination activity of each preparation of VLP was determined by serially diluting volumes of VLPs and adding an equal volume of 0.8% turkey red blood cells (RBCs) (Lampire Biologicals, Pipersville, PA, USA) suspended in PBS to a V-bottom 96-well plate with a 30 min incubation at room temperature (RT). Prepared RBCs were stored at 4°C and used within 72 h. The highest dilution of VLP with full agglutination of RBCs was considered the endpoint HA titer. The H2 HA sequences used for VLPs were Mal/NL/01, Chk/Pots/84, Muskrat/Russia/63/2014 (Musk/Rus/14) (clade-1), Duck/Cambodia/419W12M3/2013 (Duk/Cam/13) (clade-2), Japan/305/1957 (J/57) (clade-2), Moscow/1019/1965 (Mosc/65) (clade-2), T/64, Duk/HK/78, Mal/WI/08, Sw/MO/06, Quail/Rhode Island/16-018622-1/2016 (Qu/RI/16) (clade-3), and Turkey/California/1797/2008 (Tk/CA/08) (clade-3).

### Ferret vaccination and challenge experiments.

Fitch ferrets (Mustela putorius furo, spayed, female, 6 to 12 months of age) were purchased certified influenza-free and descented from Triple F Farms (Sayre, PA, USA). Ferrets were pair housed in stainless steel cages (Shor-Line, Kansas City, KS) containing Sani-Chips laboratory animal bedding (P. J. Murphy Forest Products, Montville, NJ). Ferrets were provided with Teklad Global Ferret Diet (Harlan Teklad, Madison, WI) and fresh water *ad libitum*. The University of Georgia Institutional Animal Care and Use Committee approved all experiments, which were conducted in accordance with the National Research Council’s *Guide for the Care and Use of Laboratory Animals*, the Animal Welfare Act, and the CDC/NIH’s *Biosafety in Microbiological and Biomedical Laboratories* guide. Ferrets (*n = 20*) were preinfected with H1N1, or H3N2 seasonal influenza viruses or H2N2 avian influenza viruses in different orders before vaccination. These influenza viruses included the H1N1 influenza viruses Singapore/6/1986 (Sing/86) and California/07/2009 (CA/09), the H3N2 influenza viruses Sichuan/2/1987 (Sich/87) or Panama/2007/1999 (Pan/99), and the H2N2 avian influenza viruses Chk/PA/04 or Qu/RI/16, all at an infectious dose of 1e + 6 PFU in 1 ml intranasally. For the ferrets with multiple preimmune infections, ferrets were left for 60 days between each infection and before the first vaccination.

After the establishment of preimmunity by viral infection, 60 days elapsed before ferrets were vaccinated with recombinant hemagglutinin (rHA) twice with 4 weeks between vaccinations. The ferrets were vaccinated with a 1:1 ratio (500 μl total volume) of rHA diluted with phosphate-buffered saline (PBS) (15.0 μg rHA/ferret) and the emulsified oil-water adjuvant Addavax (InvivoGen, San Diego, CA, USA). The mock-vaccinated groups received only PBS and Addavax adjuvant at a 1:1 ratio (500 μl total volume) with no rHA. Each vaccination was given intramuscularly. Before vaccinations and 2 weeks after each of the vaccinations, ferrets were bled and serum was isolated from each of the samples. The blood was harvested from all anesthetized ferrets via the anterior vena cava at days 0, 14, and 42. Blood samples were incubated at room temperature for 1 h prior to centrifugation at 6,000 rpm for 10 min. The separated serum was removed and frozen at −20°C. The ferrets were infected 4 to 6 weeks after the second vaccination with the H2N3 influenza virus Swine/Missouri/4296424/2006 (Sw/MO/06). Animals were monitored daily for 10 days postinfection for clinical symptoms such as weight loss (20% *n* = 3), lethargy (*n* = 1), sneezing, dyspnea (*n* = 2), and neurological symptoms (*n* = 3). Any ferret that reached a cumulative score (n) of three was euthanized per rules set by The University of Georgia Institutional Animal Care and Use Committee. In this study, every ferret that reached humane endpoints exhibited both lethargy (*n* = 1) and dyspnea (*n* = 2) (total score of *n* = 3).

### Hemagglutination inhibition assay.

The hemagglutination inhibition (HAI) assay was used to quantify HA-specific antibodies by measuring the inhibition in the agglutination of turkey erythrocytes. The protocol was adapted from the WHO laboratory of influenza surveillance manual (49). To inactivate nonspecific inhibitors, the serum was treated with receptor-destroying enzyme (RDE) (Denka Seiken, Co., Japan) prior to being tested. Briefly, three parts RDE were added to one-part serum and incubated overnight at 37°C. RDE was inactivated by incubating at 56°C for approximately 45 min. After the incubation period, six parts PBS were added to the RDE-treated serum. RDE-treated sera were 2-fold serially diluted in V-bottom microtiter plates. An equal volume of each virus-like particle (VLP) was adjusted to approximately 8 hemagglutination units (HAU)/25 μl and was added to each well. The plates were covered and incubated at RT for 20 min before 50 μl of RBCs were allowed to settle for 30 min at RT.

The HAI titer was determined by the reciprocal dilution of the last well that contained nonagglutinated RBCs. Positive and negative serum controls were included on each plate. Seroprotection was defined as an HAI titer of ≥1:40 and seroconversion as a 4-fold increase in titer compared to baseline, as defined by the WHO to evaluate influenza vaccines (49).

### Determination of viral nasal wash titers.

The nasal washes were performed on anesthetized ferrets by washing out each of their nostrils with a total of 3 ml of PBS on days 1, 3, 5, and 7 postinfection. From each nasal wash, ∼2.0 ml was recovered. The nasal washes were aliquoted into microcentrifuge tubes and stored at −80°C. Nasal wash aliquots were thawed at RT. Once thawed, 10-fold serial dilutions of nasal washes were overlaid on MDCK cells (49). The MDCK cells were at 95 to 100% confluence at the time that the assay was performed. Nasal wash samples were incubated for 60 min at RT with agitation every 15 min. After 60 min, the serial dilutions were removed and the MDCK cells were washed with incomplete (no fetal bovine serum [FBS]) DMEM containing penicillin and streptomycin (P/S). The wash medium was removed and replaced with 1 ml of a mixture of plaque medium without FBS, TPCK-trypsin, and 1.2% avicel. Plaque medium was made using MEM medium, HEPES buffer, l-glutamine, and P/S. All of the components of the plaque medium and avicel were obtained from Thermo Fisher Scientific (Waltham, MA, USA). The MDCK cells were incubated at 37°C with 5% CO_2_ for 48 h. After 48 h, the avicel overlay was removed and the MDCK cells were fixed with 10% buffered formalin for a minimum of 15 min. The formalin was then discarded, and the MDCK cells were stained using 1% crystal violet. The MDCK cells were then washed with distilled water to remove the crystal violet. Plaques were then counted and PFU per ml titer was calculated using the number of plaques and the appropriate dilution factor.

### Neutralization assays.

The neutralization assay was used to identify the presence of virus-specific neutralizing antibodies. The protocol was adapted from the WHO laboratory of influenza surveillance manual (49). Equal amounts of serum from each ferret within a vaccination group were combined and heat-inactivated for 30 min at 56°C. MDCK cells were grown in a 96-well flat-bottom plate until they reached 95 to 100% confluence. Antibodies were diluted in half-log increments with serum-free medium and incubated with 100× 50% tissue culture infective dose (TCID_50_) for 1 h. The antibody-virus mixture was then added to the incomplete (FBS-free) DMEM-washed MDCK cells in the 96-well plate. After 2 h, the MDCK cells were washed with incomplete DMEM. Approximately 200 μl of DMEM with P/S and 2.0 μg/ml of TPCK were added to each of the 96 wells. The cell monolayers in the back-titration control wells were checked daily until cytopathic effect (CPE) had reached the majority of the 1× TCID_50_ rows. After 3 or 4 days, 50 μl of medium per well was removed and used in an HA assay to identify the presence of influenza virus. The remaining medium in each well was removed and the MDCK cells were then fixed with 10% buffered formalin for a minimum of 15 min. The formalin was then discarded and the fixed cells were washed with 1× PBS. Afterward, the MDCK cells were stained using 1% crystal violet (Thermo Fisher Scientific, Waltham, MA, USA). The MDCK cells were then washed with distilled water to remove the crystal violet. Any well having an HA activity of ≥1:2 was defined as positive for the analysis. HA activity was confirmed by >10% of CPE in wells that was positive for HA activity.

### Statistical analysis.

Statistical significance was defined as a *P* value of less than 0.05. The limit of detection for viral plaque titers was 50 PFU/ml for statistical analysis. The viral plaque titers were transformed by log_10_ for analysis. The limit of detection for HAI was <1:10 and 1:5 was used for statistical analysis. The HAI titers were transformed by log_2_ for analysis and graphing. The geometric mean titers were calculated for neutralization assays, but the log_2_ titers were used for ANOVA analysis. All error bars on the graphs represent standard mean error. ANOVAs with Dunnet’s test were used for weight loss, with a statistical significance defined as a *P* value of less than 0.05. Nasal wash titers stratified by day and preimmunity were analyzed with a one-way ANOVA with the Tukey’s honestly significant difference method to determine differences between vaccine groups. The overall performance of the vaccines was assessed through multivariate ANOVAs for main effects conducted individually for the neutralization titer, viral nasal wash titer, and HAI titer outcomes, followed by Tukey’s honestly significant difference method for adjusting for multiple comparisons. Significantly different groups per outcome were determined from the multiple comparisons. Day 7 of the nasal wash titer was not included in the ANOVA analysis since all of the observations were below the limit of detection. All of the statistical analysis for the various assays can be found in Fig. S2, Fig. S5, and Table S1 in the supplemental material.

### Amino acid sequences.

The amino acid sequences for the two COBRA HA sequences have been reported in the United States provisional patent filing 14332088_1.

10.1128/mSphere.00052-21.1 FIG S1 Amino acid diversity in antigenic sites of WT and COBRA H2 HA sequences. The amino acid differences for the WT and COBRA H2 HA sequences in the six H2 HA antigenic sites are shown in the six tables. Amino acids are numbered based on the H3 numbering system. Only amino acid positions with differences are shown. All of the other amino acids in the antigenic sites are the same for all of the H2 HA sequences used in this study. Download FIG S1, TIF file, 0.3 MB. Copyright © 2021 Reneer et al. 2021 Reneer et al. https://creativecommons.org/licenses/by/4.0/ This content is distributed under the terms of the Creative Commons Attribution 4.0 International license.

10.1128/mSphere.00052-21.2 FIG S2 Main effects of vaccine received, established preimmunity, and day postinfection on the log 10 viral nasal wash titer. ANOVA (outcome = titer; predictors = vaccine plus preimmunity plus day) adjusted by Tukey’s honestly significant difference (HSD) method for the effect sizes (i.e., the difference between the means) for vaccines (A), preimmunity (B), and day (C) when controlling for the main effects of the other variables. The horizontal line at 0.0 indicates identical means with no measured difference. If the 95% confidence intervals extended over this line, the difference between the two compared groups are not significant at the *P* = 0.05 level. Comparisons are colored based on the adjusted *P* value. Significance groups were determined from the effect size plots for the vaccine received (D), preimmunity (E), and day (F). Groups that share a letter were not significantly different from one another. Download FIG S2, TIF file, 0.5 MB. Copyright © 2021 Reneer et al. 2021 Reneer et al. https://creativecommons.org/licenses/by/4.0/ This content is distributed under the terms of the Creative Commons Attribution 4.0 International license.

10.1128/mSphere.00052-21.3 FIG S3 Change in HAI titers from prime to boost vaccination. Columns are divided by vaccine group, and rows are divided by preimmunity. Data points from day 14 to day 42 are paired based on ferrets change in HAI titer for a virus in the HAI panel. Significance between groups are analyzed in Table S1. Download FIG S3, TIF file, 0.6 MB. Copyright © 2021 Reneer et al. 2021 Reneer et al. https://creativecommons.org/licenses/by/4.0/ This content is distributed under the terms of the Creative Commons Attribution 4.0 International license.

10.1128/mSphere.00052-21.4 FIG S4 Main effects of vaccine received, established preimmunity, and virus tested on the log 2 HAI titer on day 42. ANOVA (outcome = titer; predictors = vaccine plus preimmunity plus virus) adjusted by Tukey’s HSD method for the effect sizes (i.e., the difference between the means) for vaccines (A), preimmunity (B), and virus (C) when controlling for the main effects of the other variables. The horizontal line at 0.0 indicates identical means with no measured difference. If the 95% confidence intervals extended over this line, the difference between the two compared groups are not significant at the *P* = 0.05 level. Comparisons are colored based on the adjusted *P* value. Significance groups were determined from the effect size plots for the vaccine received (D), preimmunity (E), and virus (F). Groups that share a letter are not significantly different compared to one another. Download FIG S4, TIF file, 0.8 MB. Copyright © 2021 Reneer et al. 2021 Reneer et al. https://creativecommons.org/licenses/by/4.0/ This content is distributed under the terms of the Creative Commons Attribution 4.0 International license.

10.1128/mSphere.00052-21.5 FIG S5 Main effects of vaccine received, established preimmunity, and virus tested on the log 2 neutralization titer with pooled sera collected on day 42. ANOVA (outcome = titer; predictors = vaccine plus preimmunity plus virus) adjusted by Tukey’s HSD method for the effect sizes (i.e., the difference between the means) for vaccines (A), preimmunity (B), and virus (C) when controlling for the main effects of the other variables. The horizontal line at 0.0 indicates identical means with no measured difference. If the 95% confidence intervals extended over this line, the difference between the two compared groups are not significant at the *P* = 0.05 level. Comparisons are colored based on the adjusted *P* value. Significance groups were determined from the effect size plots for the vaccine received (D), preimmunity (E), and virus (F). Groups that share a letter are not significantly different compared to one another. Download FIG S5, TIF file, 0.8 MB. Copyright © 2021 Reneer et al. 2021 Reneer et al. https://creativecommons.org/licenses/by/4.0/ This content is distributed under the terms of the Creative Commons Attribution 4.0 International license.

10.1128/mSphere.00052-21.6 FIG S6 Establishment of preimmunity in ferrets. HAI titers for preimmune ferrets against the strains that were used to establish their influenza virus preimmunity. Serum from each ferret was obtained on day 60 postinfection and tested against the listed viruses for each preimmune group. Download FIG S6, TIF file, 0.9 MB. Copyright © 2021 Reneer et al. 2021 Reneer et al. https://creativecommons.org/licenses/by/4.0/ This content is distributed under the terms of the Creative Commons Attribution 4.0 International license.

10.1128/mSphere.00052-21.7 FIG S7 Viral nasal wash titers for H3N2-H1N1 and H1N1-H3N2 preimmune groups. Nasal washes were performed on days 1, 3, 5, and 7 postinfection. The titers are recorded as log 10 PFU/ml. The H3N2-H1N1 preimmune ferrets are shown in panels A to D. The H1N1-H3N2 preimmune ferrets are shown in panels E to H. The height of the bars shows the mean, while the error bars represent mean standard error. Download FIG S7, TIF file, 0.7 MB. Copyright © 2021 Reneer et al. 2021 Reneer et al. https://creativecommons.org/licenses/by/4.0/ This content is distributed under the terms of the Creative Commons Attribution 4.0 International license.

10.1128/mSphere.00052-21.8 TABLE S1 Paired *t* tests of log 2 HAI titers measured after prime (day 14) and boost (day 42) vaccinations. The Holm correction was used to adjust for multiple comparisons. Samples were paired based on the ferret. The *t* tests were conducted to determine if HAI titers changed after stratification by preimmunity and vaccine received. * *P* < 0.05; ** *P* < 0.01; *** *P* < 0.001. Download Table S1, TIF file, 0.3 MB. Copyright © 2021 Reneer et al. 2021 Reneer et al. https://creativecommons.org/licenses/by/4.0/ This content is distributed under the terms of the Creative Commons Attribution 4.0 International license.
