# Supplementary figures and images for: Broadly Reactive H2 Hemagglutinin Vaccines Elicit Cross-Reactive Antibodies in Ferrets Preimmune to Seasonal Influenza A Viruses
Source: mSphere. 2021 Mar 10;6(2):e00052-21. doi: 10.1128/mSphere.00052-21 (PMC8546680; doi:10.1128/mSphere.00052-21)

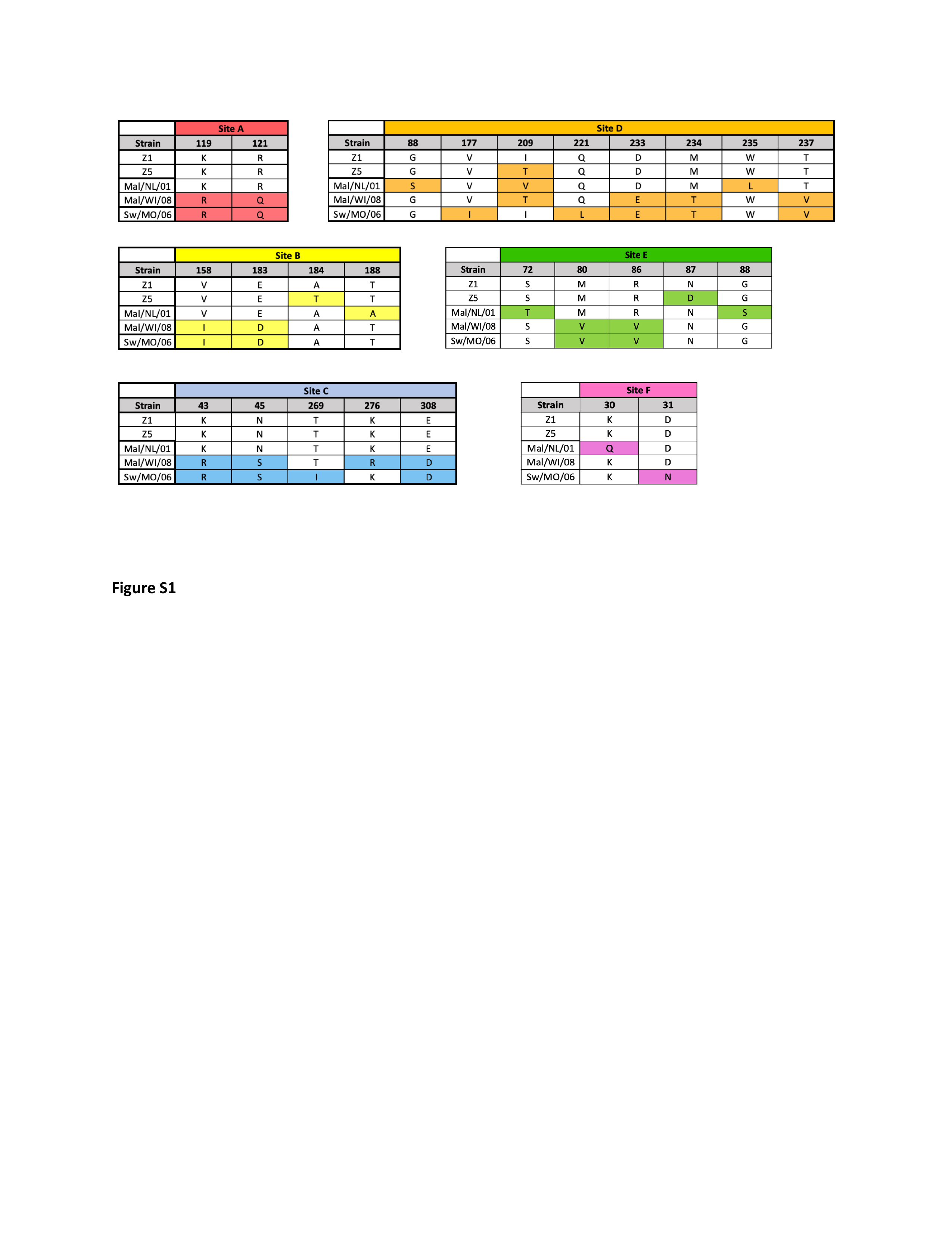

Supplement: FIG S1 [file msphere.00052-21-sf001.tif]

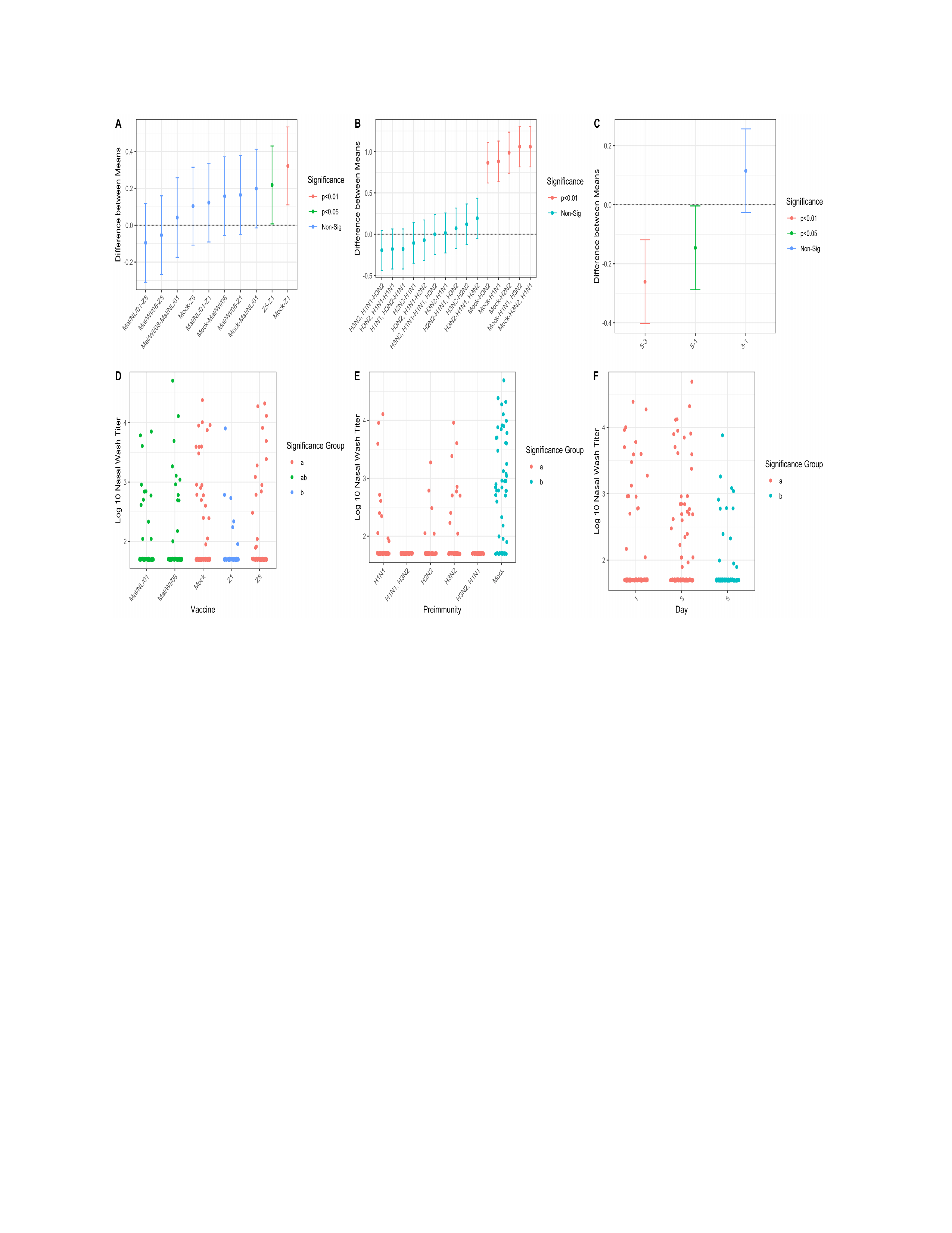

Supplement: FIG S2 [file msphere.00052-21-sf002.tif]

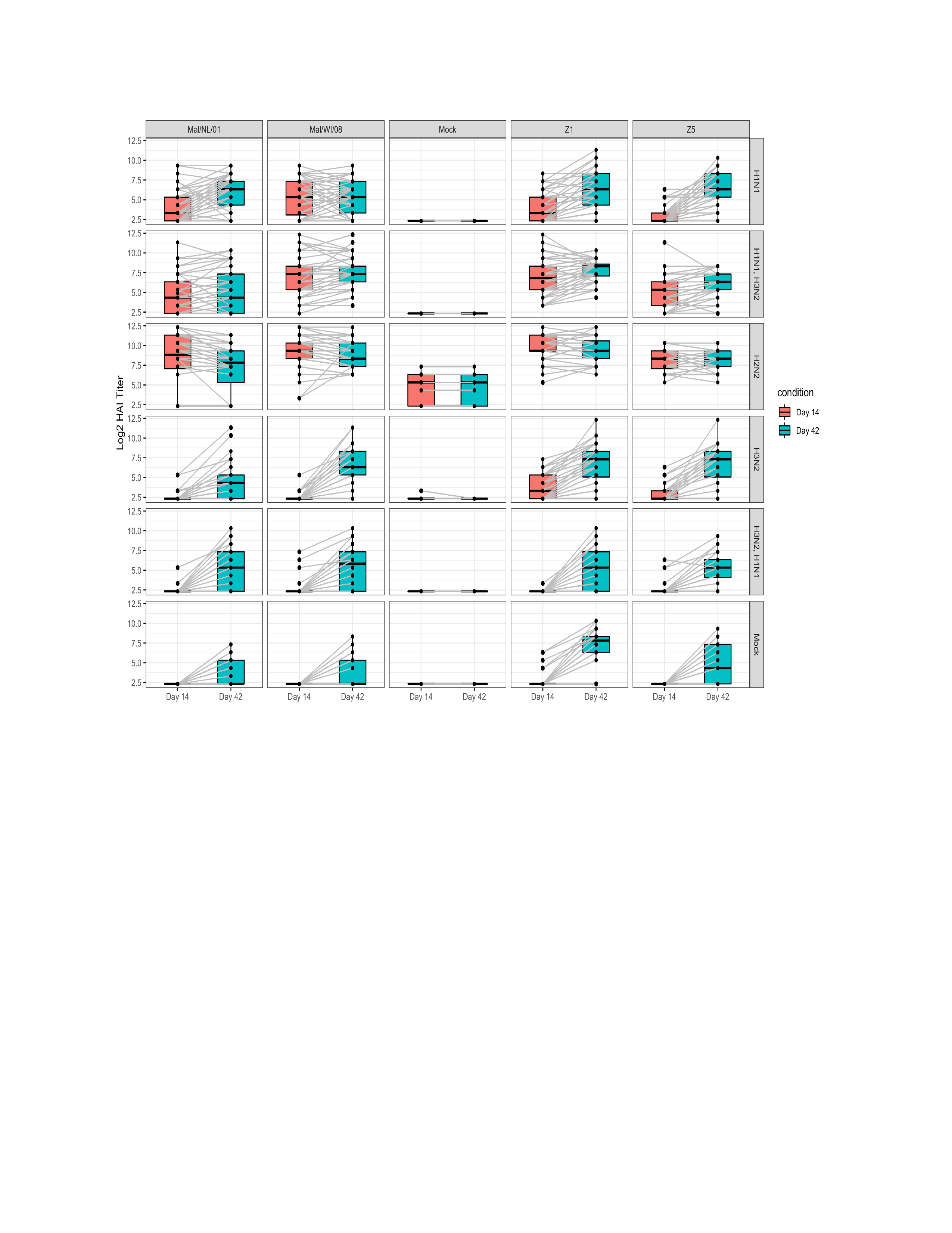

Supplement: FIG S3 [file msphere.00052-21-sf003.tif]

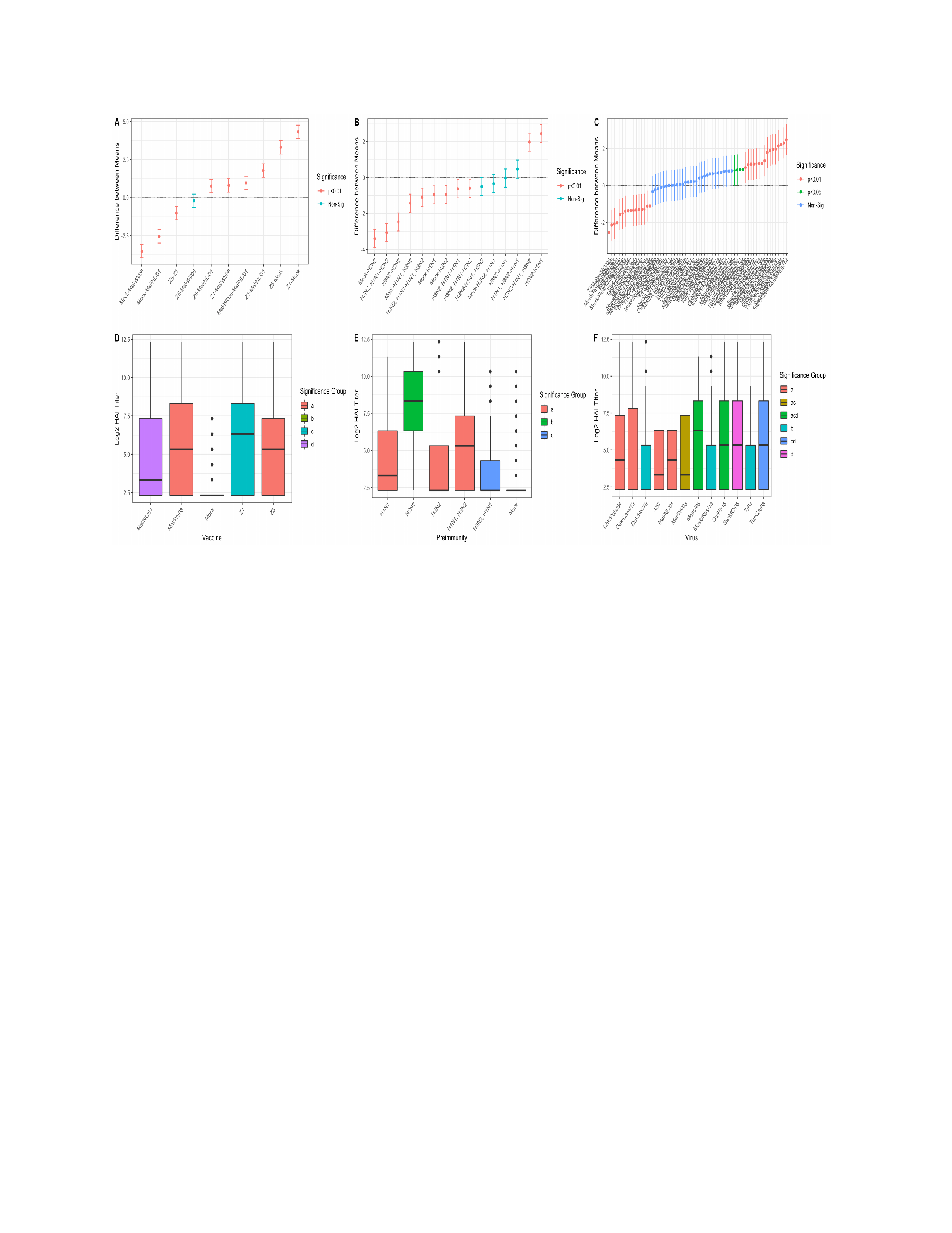

Supplement: FIG S4 [file msphere.00052-21-sf004.tif]

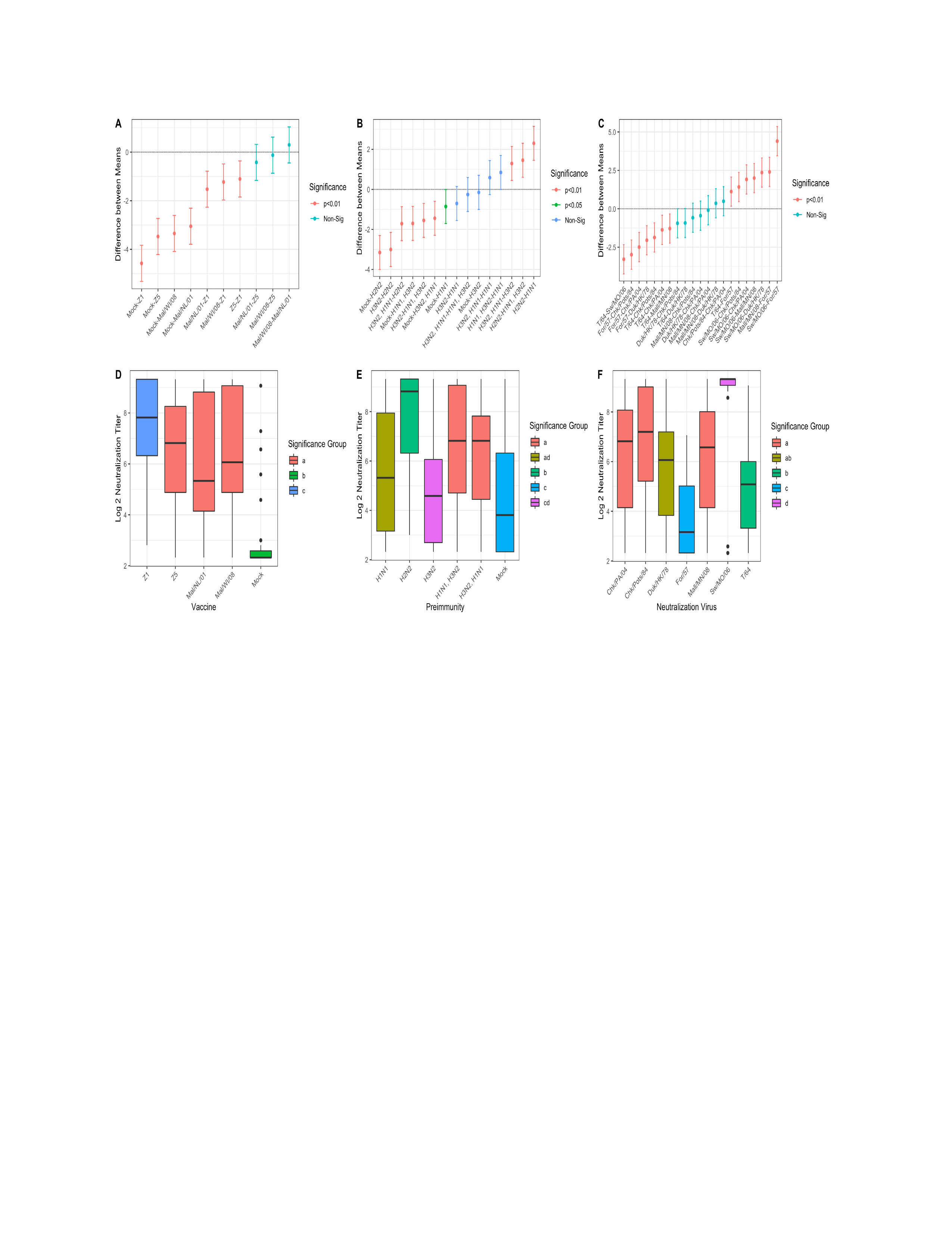

Supplement: FIG S5 [file msphere.00052-21-sf005.tif]

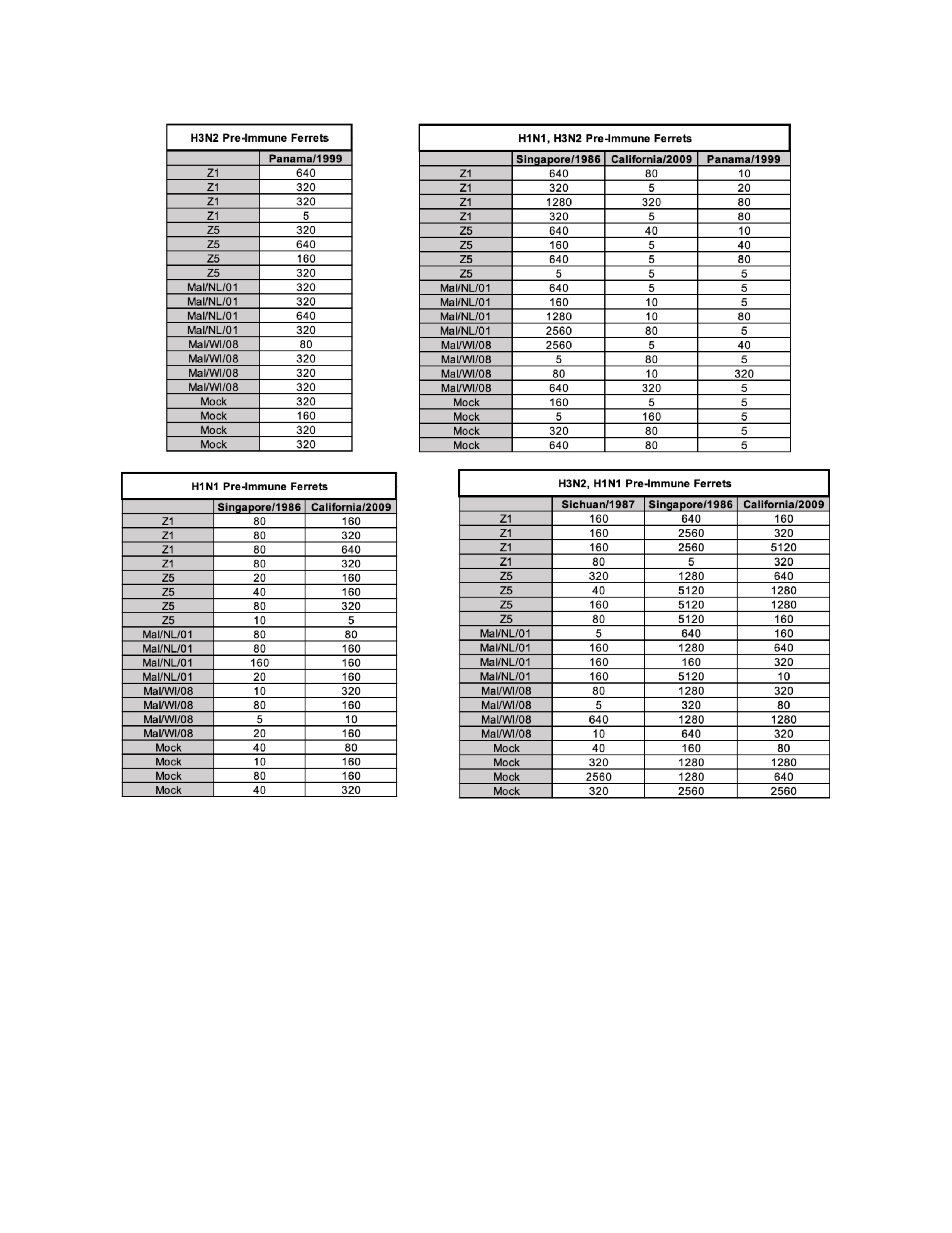

Supplement: FIG S6 [file msphere.00052-21-sf006.tif]

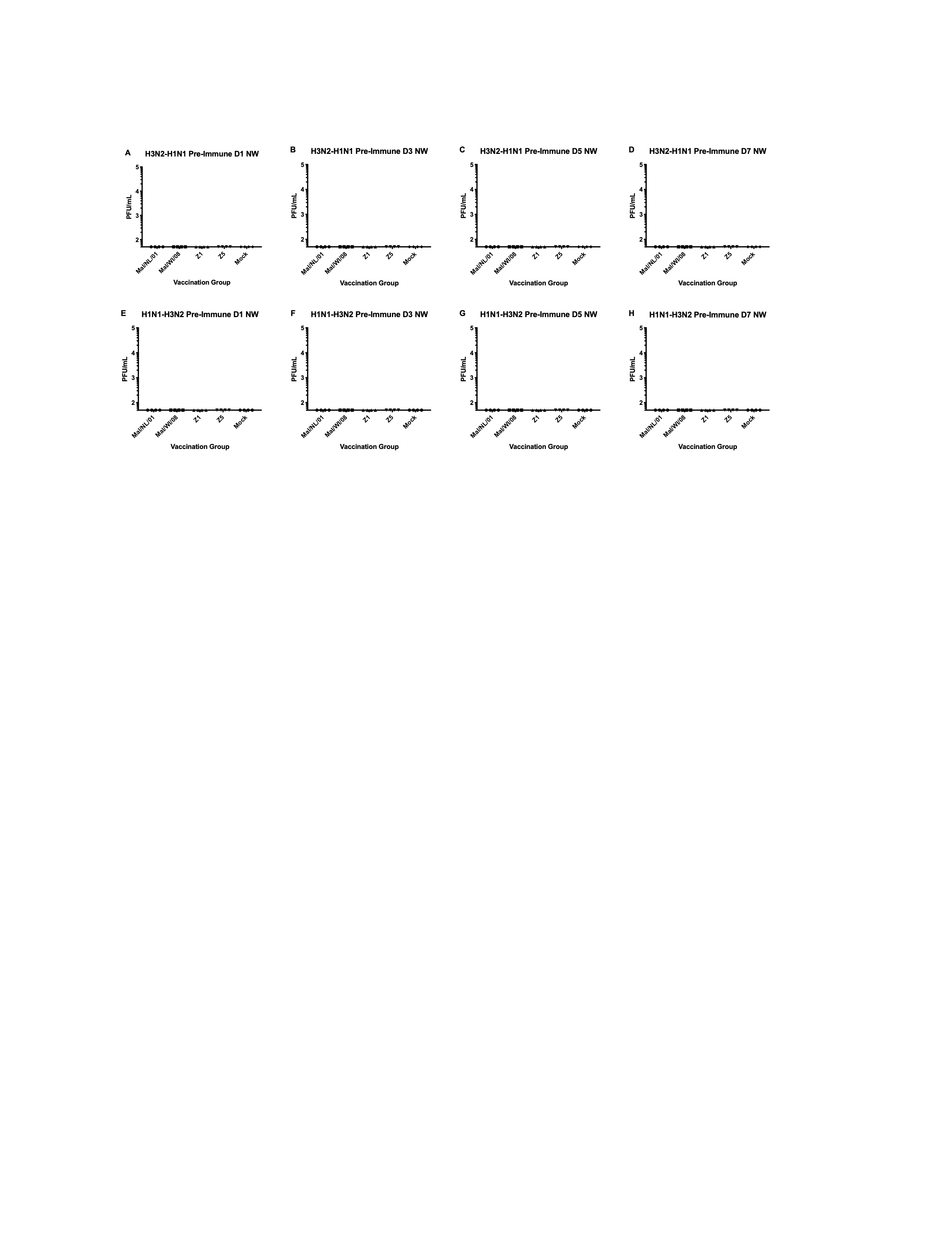

Supplement: FIG S7 [file msphere.00052-21-sf007.tif]

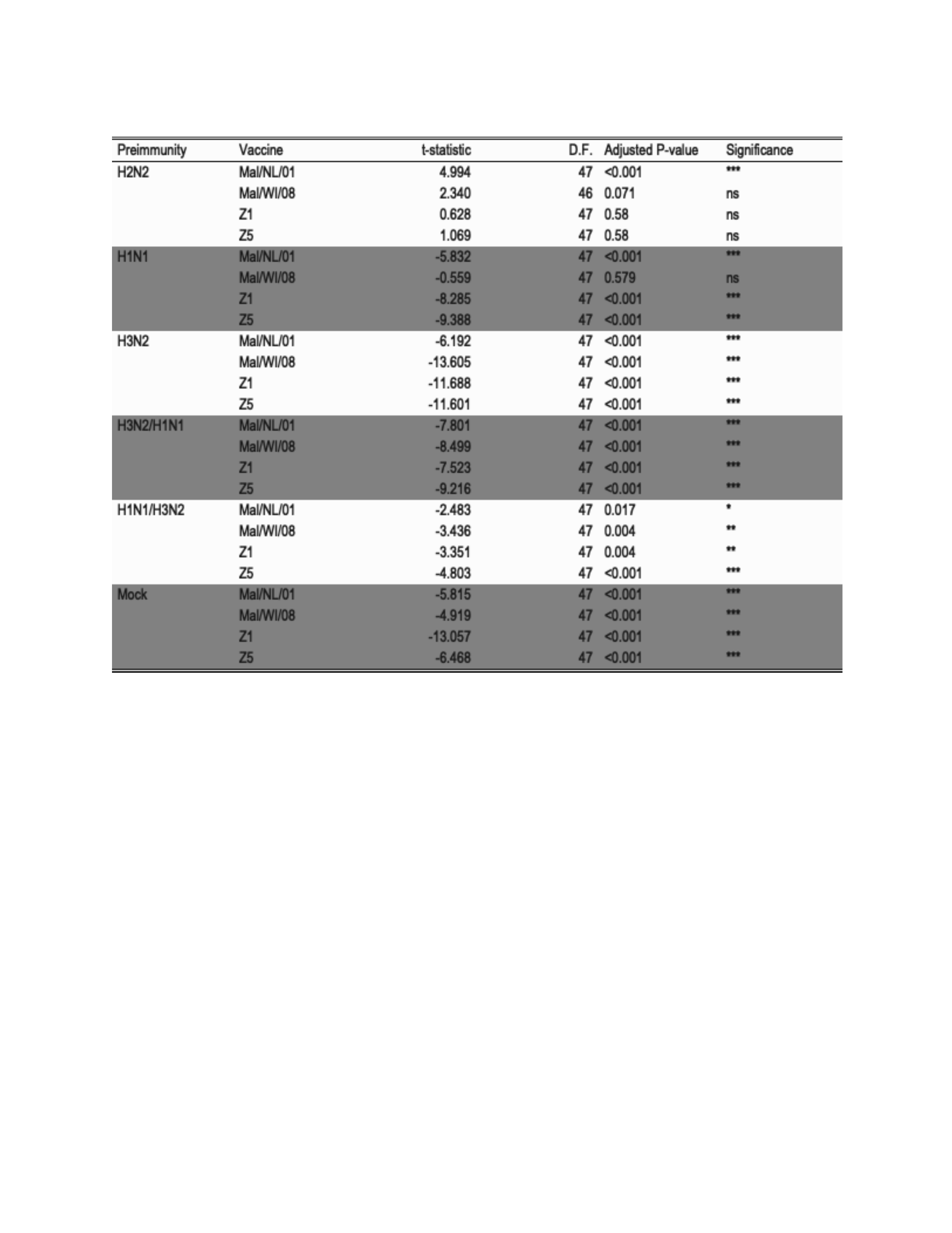

Supplement: TABLE S1 [file msphere.00052-21-st001.tif]
